# Tethered TGF-β1 in a Hyaluronic Acid-Based Bioink for Bioprinting Cartilaginous Tissues

**DOI:** 10.3390/ijms23020924

**Published:** 2022-01-15

**Authors:** Julia Hauptstein, Leonard Forster, Ali Nadernezhad, Jürgen Groll, Jörg Teßmar, Torsten Blunk

**Affiliations:** 1 Department of Trauma, Hand, Plastic and Reconstructive Surgery, University of Würzburg, 97080 Würzburg, Germany; hauptstein_j@ukw.de; 2 Department for Functional Materials in Medicine and Dentistry, Bavarian Polymer Institute (BPI), University of Würzburg, 97070 Würzburg, Germany; leonard.forster@fmz.uni-wuerzburg.de (L.F.); ali.nadernezhad@fmz.uni-wuerzburg.de (A.N.); juergen.groll@fmz.uni-wuerzburg.de (J.G.); joerg.tessmar@fmz.uni-wuerzburg.de (J.T.)

**Keywords:** biofabrication, bioink, chondrogenic differentiation, dual-stage crosslinking, hyaluronic acid, tethering, transforming growth factor-beta 1

## Abstract

In 3D bioprinting for cartilage regeneration, bioinks that support chondrogenic development are of key importance. Growth factors covalently bound in non-printable hydrogels have been shown to effectively promote chondrogenesis. However, studies that investigate the functionality of tethered growth factors within 3D printable bioinks are still lacking. Therefore, in this study, we established a dual-stage crosslinked hyaluronic acid-based bioink that enabled covalent tethering of transforming growth factor-beta 1 (TGF-β1). Bone marrow-derived mesenchymal stromal cells (MSCs) were cultured over three weeks in vitro, and chondrogenic differentiation of MSCs within bioink constructs with tethered TGF-β1 was markedly enhanced, as compared to constructs with non-covalently incorporated TGF-β1. This was substantiated with regard to early TGF-β1 signaling, chondrogenic gene expression, qualitative and quantitative ECM deposition and distribution, and resulting construct stiffness. Furthermore, it was successfully demonstrated, in a comparative analysis of cast and printed bioinks, that covalently tethered TGF-β1 maintained its functionality after 3D printing. Taken together, the presented ink composition enabled the generation of high-quality cartilaginous tissues without the need for continuous exogenous growth factor supply and, thus, bears great potential for future investigation towards cartilage regeneration. Furthermore, growth factor tethering within bioinks, potentially leading to superior tissue development, may also be explored for other biofabrication applications.

## 1. Introduction

In recent years, 3D biofabrication including bioprinting has evolved as a fast-growing research field in regenerative medicine and for the development of disease models [1,2,3]. 3D bioprinting enables precise patterning of cells and hydrogel materials, i.e., bioinks, and is investigated as a promising alternative approach for treatment of cartilage defects in trauma and degenerative diseases. To obtain functional cartilage transplants, bioinks that support chondrogenic development are of key importance [4,5,6,7].

Hyaluronic acid represents a promising and attractive material for cartilage regeneration, as it is a main component of the natural cartilage extracellular matrix (ECM), and it enables diverse chemical modifications for crosslinking reactions with other bioink components [4,8,9,10]. Frequently applied functionalizations are, for example, thiol [11], methacrylate [12], glycidyl methacrylate [13], tyramine [14], or norbornene [15] among several others. Nevertheless, studies that show flexible hyaluronic acid inks with a high initial shape stability after printing as well as convincing long-term development of cartilaginous constructs are still rare [8,16].

Apart from a suitable ink material, the growth factor TGF-β is crucial for chondrogenic differentiation of mesenchymal stromal cells (MSCs) and cartilage homeostasis. It stimulates ECM production and can enhance cartilage repair, while its absence results in osteoarthritis (OA)-like changes [17,18]. However, application of TGF-β to the synovial tissue of the joint, for example via injections, results in a short half-life [19] and can induce fibrosis and osteophyte formation in adjacent tissues [20,21]. Therefore, a precise local supplementation and dose control appears desirable. TGF-β immobilization within hydrogel scaffolds could be achieved, e.g., by direct loading, encapsulation into particulate carriers which are incorporated into the scaffold, reverse binding, for example via electrostatic interactions, or covalent tethering [22,23]. Especially the covalent approach holds great promise for functional cartilage transplants with respect to prolonged availability for embedded MSCs and low release rates into the surrounding tissue [24,25,26]. With regard to 3D bioprinting, covalent incorporation into the used bioink may avert the necessity for repeated injections in vivo, likely enhancing the clinical potential of the inks for cartilage regeneration. Over the last couple of years, the impact of shear stress during 3D printing on cell survival and differentiation has been extensively studied [27,28,29,30]. Furthermore, it is known that shear stress can also strongly impact protein structure and function [31,32]. However, surprisingly, studies that investigate whether the 3D printing process may have an impact on the functionality of covalently tethered growth factors are still lacking. For this reason, this study focused on the protein function of soluble and covalently tethered TGF-β1 in a comparative analysis of cast and printed bioinks.

In a recent study, we established a novel hyaluronic acid-based bioink platform for cartilage 3D biofabrication [16]. Within this platform, we identified bioinks which enabled high shape fidelity during and after printing, but still had a very low polymer content of 2% and a highly porous network. This facilitated effective chondrogenic differentiation of MSCs, a homogeneous distribution of newly produced extracellular matrix (ECM) and thereby a superior quality of resulting cartilaginous constructs. However, these novel bioinks were not suitable for growth factor binding and TGF-β1 had to be supplemented with each medium change.

Therefore, in this study, we investigated a new hyaluronic acid-based bioink which maintained the beneficial properties of the established bioink platform and further enabled covalent TGF-β1 tethering. Specifically, TGF-β1 was thiol-modified with Traut’s reagent and covalently tethered to a newly integrated crosslinker, polyethylene glycol-octaacrylate (8-arm PEG-acryl), via Michael addition. In the first step of hydrogel formation, the remaining acryl groups of 8-arm PEG-acryl reacted via Michael addition with thiol-modified hyaluronic acid (HA-SH), resulting in a highly viscous and 3D printable ink. After fabrication, a UV-induced thiol-ene click reaction between polyethylene glycol-diallyl carbamate (2-arm PEG-allyl) and the residual thiol groups of HA-SH ensured final crosslinking. We characterized the inks with regard to rheological properties, swelling behavior, and TGF-β1 release. MSC chondrogenesis was assessed in cast constructs with varying amounts of covalently or non-covalently incorporated TGF-β1, with distinct advantages achieved by TGF-β1 tethering. Subsequently, constructs with 150 nm TGF-β1, either tethered or non-covalently incorporated, were cast or 3D bioprinted. Chondrogenic differentiation of MSCs was extensively evaluated regarding early TGF-β1 signaling, gene expression, qualitative and quantitative ECM deposition and distribution, and resulting construct stiffness. The presented HA-based bioink with tethered TGF-β1 yielded superior chondrogenic differentiation, as compared to constructs with non-covalently incorporated TGF-β1. Furthermore, it was successfully demonstrated in the comparative analysis of cast and printed bioinks that covalently tethered TGF-β1 maintained its functionality after 3D printing.

## 2. Results and Discussion

### 2.1. Bioink Composition and Dual-Stage Crosslinking

This study presents a novel 3D printable hyaluronic acid-based bioink that allows for biofunctionalization with tethered TGF-β1 to generate advanced cartilaginous 3D constructs without the need for an exogenous supply of the differentiation factor during long-term culture. Previously, TGF-β1 has been bound to hydrogels via Traut’s reagent and other covalent approaches to induce differentiation and ECM production of embedded MSCs or chondrocytes, however, only in non-printable hydrogels [24,25,26,33,34,35]. The HA-based ink presented here utilized a recently established dual-stage crosslinking mechanism [16], with adapted composition in order to facilitate TGF-β1 tethering. Ink components encompassed thiol-modified hyaluronic acid (HA-SH, 465 kDa), acryl-modified polyethylene glycol (8-arm PEG-acryl, 10 kDa), and allyl-modified PEG (2-arm PEG-allyl, 6 kDa). Successful synthesis of all ink components was verified via GPC and ^1^H-NMR (Figure S1, Supplementary Materials). For the newly established 8-arm PEG-acrylate it could be demonstrated that the acrylation was successful and quantitative using the enzymatic catalysis and no oligomers were obtained during modification (Figure S1b, Supplementary Materials).

**Figure 1:**
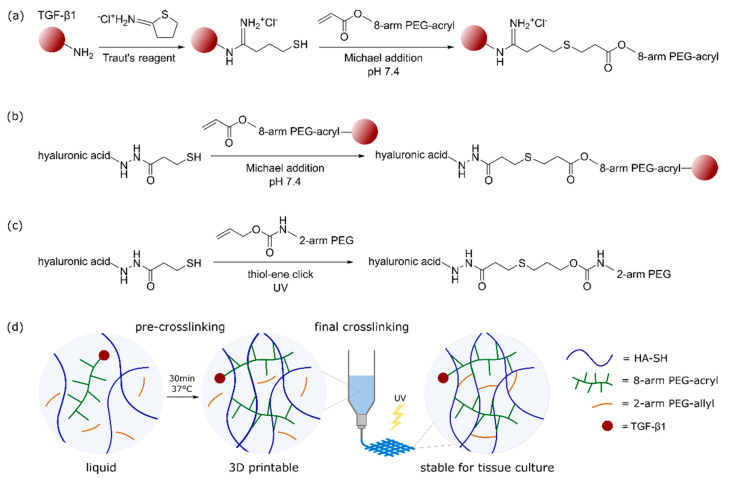
Ink crosslinking mechanism and functionalization with TGF-β1. (**a**) Thiol-functionalization of TGF-β1 with Traut’s reagent and Michael addition with 8-arm PEG-acryl. (**b**) Michael addition of TGF-β1-modified PEG-acryl with HA-SH (pre-crosslinking), resulting in a 3D printable ink. (**c**) UV-induced final crosslinking of residual HA-SH thiol groups with 2-arm PEG-allyl in the presence of Irgacure I2959 at 365 nm. (**d**) Graphical overview of the dual-stage crosslinking mechanism and workflow of TGF-β1 tethered construct generation for chondrogenic tissue culture.

Figure 1 shows the detailed functionalization and crosslinking mechanism of the used ink composition. At first, free lysine residues of TGF-β1 were thiol-modified using Traut’s reagent in a 4:1 molar excess of Traut to TGF-β1, as described previously [24,25,26]. Subsequently, 8-arm PEG-acryl was added to react with thiol-modified TGF-β1 in a spontaneous Michael addition at pH 7.4 (Figure 1a). In the next step of bioink formulation, all other ink components (HA-SH, 2-arm PEG-allyl, I2959) as well as human MSCs (20 × 10^6^ mL^−1^) were mixed with the TGF-β1-functionalized PEG-acryl solution. Within 30 min of incubation at 37 °C, free acryl groups of PEG-acryl reacted in a Michael addition at pH 7.4 with the main fraction of HA-SH thiol groups to generate a highly viscous and printable bioink (Figure 1b). After 3D printing, constructs were UV-irradiated at 365 nm to finally crosslink the residual HA-SH thiol groups with allyl groups of PEG-allyl via thiol-ene click chemistry in the presence of the photoinitiator Irgacure I2959 (Figure 1c). Figure 1d presents a schematic graphical overview to visualize the ink composition with its two different crosslinking processes. The used ink had an overall polymer content of 1.4% (0.5% HA-SH, 0.5% 8-arm PEG-acryl, 0.4% 2-arm PEG-allyl and 0.05% I2959; all *w*/*v*), and still enabled stand-alone printability. Such low polymer content has previously been shown to be favorable with regard to diffusion properties and penetrability of cellularly produced ECM in non-printed [36,37] and printed constructs [16,38].

### 2.2. 3D Printing and Ink Characterization

We have previously characterized printability of dual-stage crosslinked platform bioinks in detail [16]. In order to prove printability of the adapted ink with the new component 8-arm PEG-acrylate presented in this study, filament fusion tests and strand thickness evaluations were performed accordingly (Figure S2a,b, Supplementary Materials). Filaments fused at the smallest distance of 0.5 mm and partially at 0.75 mm, strand thickness measurements yielded the average strand thickness of 650 µm and a strand intersection diagonal of around 1.1 mm, which was well in accordance with the previously reported results of the printable platform inks [16]. Additionally, as a proof-of-principle, a large construct with 21 layers was 3D printed, resembling human femoral condyles (Figure S2c, Supplementary Materials).

**Figure 2:**
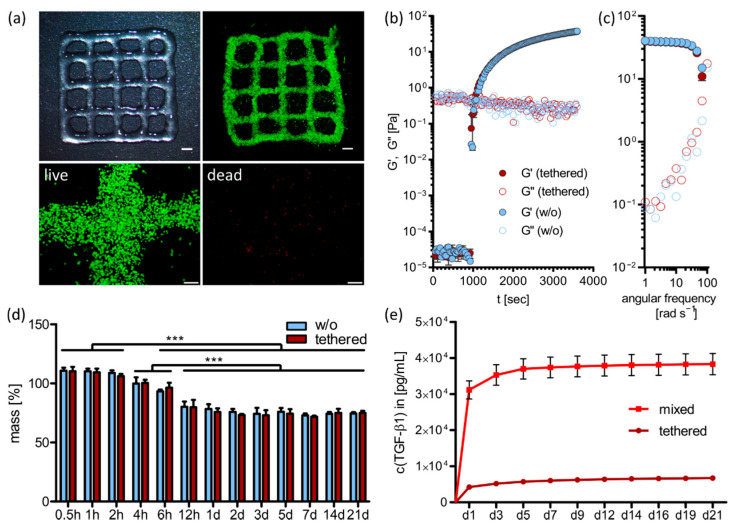
3D printing and ink characterization. (**a**) Overview of 3D printed grids and survival of MSCs after printing. Living cells are labeled with calcein-AM (green) and dead cells with EthD-III (red). Scale Bars of overview images represent 2 mm, scale bars of split channel images at higher magnification represent 200 µm. (**b**) Time sweep and (**c**) frequency sweep measurements of the pre-crosslinking reaction (Michael addition) showing the progression and gelation state of inks with 150 nm tethered TGF-β1 or without the protein. (**d**) Swelling analysis of constructs without or with 150 nm tethered TGF-β1 over three weeks. Data are represented as the percentage deviation from the original wet weight (=100%) as mean ± standard deviation (*n* = 3). Significant differences are marked with *** (*p* < 0.001). (**e**) TGF-β1 release from constructs with 150 nm tethered or mixed TGF-β1 over 3 weeks analyzed by ELISA (*n* = 3). Tested conditions are significantly different at all time points with *p* < 0.001.

Furthermore, we analyzed biocompatibility of the bioink and possible physical impacts of TGF-β1 tethering on bioink and construct properties. Therefore, the bioink was prepared as described above and printed in 2-layered constructs to enable imaging with a low background signal and quantification of single cells. Figure 2a shows an overview of the printed grid structures and survival of MSCs directly after printing. To visualize living and dead cells separately, the split channels of a representative image with higher magnification is shown in the second row. Quantification of overall cell survival yielded around 98% living cells and only 2% dead cells after printing. This proved well-adapted printing conditions and confirmed the bioink as a suitable cell carrier.

To assess the potential influence of covalent TGF-β1 tethering to 8-arm PEG-acryl on the gelation and, eventually, viscoelastic properties of the ink, we evaluated the kinetics of the first stage of crosslinking (i.e., the Michael addition). Rheological characterization of time and frequency sweeps were performed on samples without the protein or with 150 nm tethered TGF-β1 (Figure 2b,c). Time sweep measurements revealed a drastic increase in ink viscosity after 15 min and almost complete Michael addition after 30 min. This corresponded to empirically defined pre-crosslinking times of this ink in 3D printing experiments for cell culture. Furthermore, covalent incorporation of TGF-β1 did not affect the mobility and reactivity of 8-arm PEG-acryl, and the kinetics of progression of Michael addition was almost identical in both formulations (i.e., without or with tethered TGF-β1). Moreover, the results of frequency sweep experiments showed that after the same incubation time at 37 °C, the formed covalent bonds during Michael addition yielded a similar state of long-range interactions between polymer chains. These results demonstrated that the presence of covalently bound TGF-β1 did not compromise gelation during the first stage of crosslinking.

For further construct characterization, we examined gel stability after final crosslinking for constructs without or with 150 nm tethered TGF-β1 (Figure 2d). Therefore, ink was prepared without cells, cast in a defined cylindrical geometry (5 mm diameter, 40 µL volume), and incubated in PBS over three weeks. No significant differences between the constructs with or without tethered TGF-β1 could be detected at all time points (0.5, 1, 2, 4, 6, 12 h, 1, 2, 3, 5, 7, 14, 21 d). Both gel systems initially swelled slightly to 110% within the first two hours and then started to shrink to around 80%, reaching their equilibrium state at 12 h after preparation. The swelling process was due to initial water inflow into the hydrogel system and shrinkage may be explained by formation of disulfide bonds between unreacted HA-SH thiol groups. Statistically, all thiol groups should be saturated by PEG-acryl or PEG-allyl crosslinking, but it could not be excluded that some functional groups were not in close proximity to react with each other and accordingly thiols remained available for disulfide formation.

In general, no significant impact of TGF-β1 tethering was detected on ink viscosity or the stability of the crosslinked constructs. Calculations of present equivalents of the reacting compounds during covalent tethering of 150 nm TGF-β1 to the used bioink revealed that one modified TGF-β1 molecule was facing 3.3 × 10^3^ PEG-acryl molecules. As the used PEG-acryl crosslinker has eight functional groups per molecule, this means that only approximately every 400th acryl group of the PEG crosslinkers was functionalized with TGF-β1. Accordingly, no impact on ink crosslinking mechanisms and resulting construct stability was expected, even for the highest growth factor concentration investigated.

To further characterize protein tethering within the bioink, we analyzed TGF-β1 release from constructs with 150 nm tethered TGF-β1 (with Traut’s reagent) and non-covalently bound TGF-β1 (mixed without Traut’s reagent). Therefore, gels without cells were incubated in 1% BSA/PBS over 21 days and TGF-β1 concentration in the medium was examined via ELISA (Figure 2e). A burst release was detected for non-covalently bound TGF-β1 (mixed) within the first day and a cumulative release of 3.8 × 10^4^ pg mL^−1^ after three weeks. In contrast, only 6.7 × 10^3^ pg mL^−1^ diffused out of the constructs with covalently tethered TGF-β1 within the same time. This corresponded to 25.6% (mixed) and 4.5% (tethered) of initially applied protein, respectively. Hyaluronic acid contains many hydrophilic groups that can interact via electrostatic forces with positively charged amino acid residues on growth factors [39,40,41,42], which might be the reason for the relatively low protein release of 25.6% in the mixed group. However, significantly lower TGF-β1 release of 4.5% was observed in the tethered group confirming effective growth factor thiol-functionalization with Traut’s reagent and PEG-acryl tethering via Michael addition.

### 2.3. Impact of TGF-β1 Concentration and Administration on MSC Differentiation

**Figure 3:**
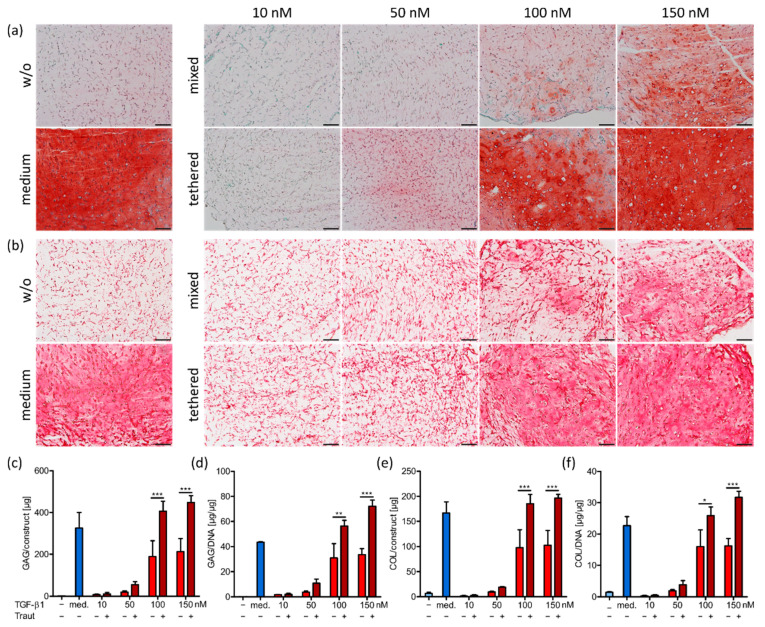
Histological staining and quantification of ECM components in cast constructs after 21 days. (**a**) Staining for GAG (safranin O) and (**b**) collagen (picrosirius red) in constructs with 10, 50, 100 or 150 nm mixed (−Traut) or tethered (+Traut) TGF-β1, as well as control groups cultured without (w/o, −) or with TGF-β1 (med.) as medium supplement. Scale bars represent 100 µm. (**c**) Quantification of glycosaminoglycan (GAG) content in the constructs and (**d**) normalized to DNA. (**e**) Quantification of collagen (COL) content in the constructs and (**f**) normalized to DNA. Data are represented as mean ± standard deviation (*n* = 3). Significant differences are marked with * (*p* < 0.05), ** (*p* < 0.01) and *** (*p* < 0.001).

After successful material characterization, we analyzed the chondrogenic potential of the biofunctionalized ink. To examine the optimal growth factor concentration for chondrogenic differentiation of human MSCs, constructs were prepared and cast as described above with either covalently tethered or only mixed TGF-β1, i.e., with or without Traut’s reagent, in different concentrations (10 nm, 50 nm, 100 nm and 150 nm) and cultured in a TGF-β1-free differentiation medium for 21 days. As a control, constructs without incorporated TGF-β1 were cultured in differentiation medium supplemented with 10 ng mL^−1^ TGF-β1 at each medium change, or without the supplementation as a negative control. Cell survival was very good under all conditions after construct preparation on day 1 as well as after 21 days in the respective culture conditions (Supplementary Materials, Figure S3). Glycosaminoglycans (GAG) and collagens, as the main components of natural cartilage ECM, were analyzed in the MSC-laden constructs after 21 days (Figure 3). Constructs without TGF-β1 supplementation showed no GAG (Figure 3a) and collagen (Figure 3b) production, while control constructs in medium supplemented with TGF-β1 showed strong differentiation. In the constructs with incorporated TGF-β1, the production of both GAG and collagen increased with increasing growth factor concentrations (10–150 nm), and importantly, tethered TGF-β1 induced markedly higher ECM production compared to the corresponding mixed conditions. These results were observed in histological stainings (Figure 3a,b), and further confirmed by quantification of total GAG and collagen amounts in the constructs (Figure 3c,e) as well as amounts normalized to DNA content (Figure 3d,f). The concentrations of 10 nm and 50 nm TGF-β1 (tethered (+Traut) and mixed (−Traut)) were not sufficient to induce adequate chondrogenic differentiation of MSCs. The concentrations of 100 nm and 150 nm tethered TGF-β1 allowed for high amounts of newly produced GAG and collagen, while non-tethered (mixed) TGF-β1 at 100 nm and 150 nm resulted in significantly reduced differentiation capacity.

In general, TGF-β1 concentration of 150 nm exhibited the best results regarding ECM production and distribution. Therefore, this concentration was used in the following printing studies. Constructs with 150 nm tethered TGF-β1 and control constructs in medium supplemented with TGF-β1 demonstrated that a homogeneous ECM distribution was achievable. This was likely due to the low overall polymer content of the used hydrogel composition (1.4%) and validated this ink as a beneficial cell carrier for cartilage biofabrication [36,37,43]. Concentration dependence of MSC or chondrocyte differentiation induced by TGF-β1 tethered via Traut’s reagent has been previously analyzed in not-printable hydrogels [24,25,26]. Only one study compared tethered with mixed TGF-β1 incorporation and detected very similar differences as those found in this study [24]. All presented results clearly indicated the superior effect of covalently tethered TGF-β1 into the ink compared to non-covalently incorporated growth factor (mixed). This can be partially explained by the differences in TGF-β1 release profiles between the conditions (Figure 2e). A further explanation might be that free TGF-β1 can undergo endocytosis and degradation when bound to the cell surface receptor on MSCs, thus reducing the available protein concentration [44,45]. In contrast, covalently tethered protein might trigger prolonged signaling without potential internalization.

### 2.4. Investigation of TGF-β1 Functionality after Bioprinting

Extrusion-based bioprinting enables patterning of cells and biomaterials in a desired 3D structure, but still, incorporated cells and proteins can suffer from shear stress during the printing process [27,31,32,46,47]. Therefore, we examined TGF-β1 functionality in cast and printed bioinks with 150 nm covalently tethered TGF-β1 as well as non-covalently incorporated protein (mixed). Chondrogenic differentiation of incorporated MSCs was used as a reliable readout for protein function. As a control, we used cast and printed constructs fabricated without TGF-β1 and subsequently cultured in differentiation medium either without the protein or supplemented with 10 ng mL^−1^ soluble TGF-β1 at every medium change. To examine the influence of the printing process, bioinks were either pipetted in disk form (5 mm diameter, 40 µL volume) (cast) or first extruded at the same printing conditions used for grid printing (Figure 2a) and afterwards pipetted in the same cylindrical geometry (print). This procedure enabled final gelation in the same construct geometry to facilitate comparability and avoid the potential impact of different nutrient and oxygen supply in differently shaped constructs [38,48,49]. Cell survival was very good in all conditions on day 1 and after 21 days, without differences between cast and printed approaches (Figure S4, Supplementary Materials).

#### 2.4.1. TGF-β1 Signaling and Chondrogenic Gene Expression

**Figure 4:**
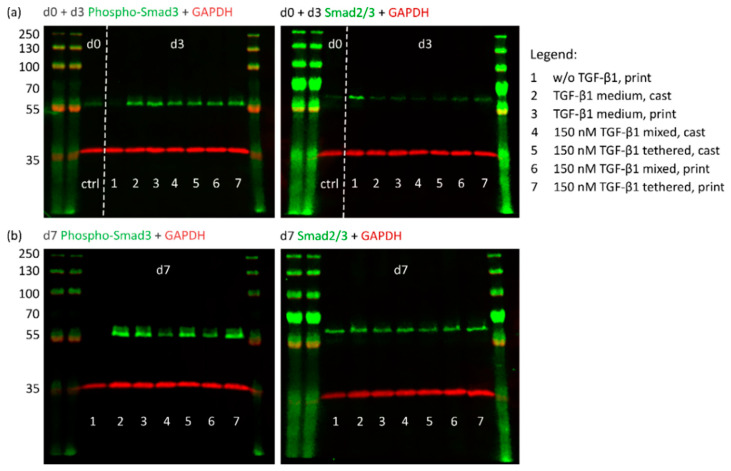
Western blot analysis of TGF-β1/Smad signaling in cast and printed constructs. Cast and printed constructs with 150 nm mixed (−Traut) or tethered (+Traut) TGF-β1 were analyzed for their protein expression. Constructs cultured without or with TGF-β1 as medium supplement served as controls. Samples were analyzed directly after fabrication (d0, ctrl) and all test conditions after (**a**) 3 and (**b**) 7 days. TGF-β1-induced active Smad signaling is shown by staining of phosphorylated Smad3 (P-Smad3) and can be compared with the unphosphorylated levels of the transcription factor (Smad2/3) (expected *M*_W_ 52 kDa). GAPDH served as loading control (expected *M*_W_ 38 kDa). The protein ladder is labeled on the left (protein *M*_W_ 35–250 kDa).

TGF-β1 induces the canonical Smad-dependent signaling cascade through binding to cell surface receptors of MSCs. Upon receptor binding, Smad2/3 is phosphorylated and translocates to the nucleus where it is involved in chondrogenic target gene expression [50,51,52]. To detect the possible impact of the printing process on TGF-β1 functionality, Smad phosphorylation was analyzed via Western blotting (Figure 4).

As a first response to TGF-β1 binding, Smad2/3 phosphorylation was expected early during culture. Furthermore, the initial burst release of unbound TGF-β1 was determined during the first three days (Figure 2e). For these reasons, we analyzed cast and printed constructs with 150 nm covalently tethered or mixed TGF-β1 for activated phospho-Smad3 (P-Smad3) proteins after 3 and 7 days of culture. As a control, constructs without TGF-β1 were harvested directly after preparation, i.e., before incubation in culture medium (d0). After 3 days, activated P-Smad3 was found in all constructs which had contact to TGF-β1, independent of application (lanes 2–7). Only the constructs without TGF-β1 (lane 1) lacked P-Smad3. Basal levels of unphosphorylated Smad2/3 were detected in all constructs, although to a distinctly lesser extent in the conditions with TGF-β1 (Figure 4a). After 7 days, medium controls (lanes 2 and 3), as well as the constructs with 150 nm covalently tethered TGF-β1 (lanes 5 and 7) contained high levels of P-Smad3, while constructs with 150 nm mixed TGF-β1 (lanes 4 and 6) showed distinctly lower concentrations (Figure 4b). Constructs without TGF-β1 (lane 1) completely lacked the activated P-Smad3 protein. Unphosphorylated Smad2/3 protein was detected evenly in all conditions. Importantly, no obvious differences in Smad phosphorylation could be determined between cast and printed constructs (Figure 4).

**Figure 5:**
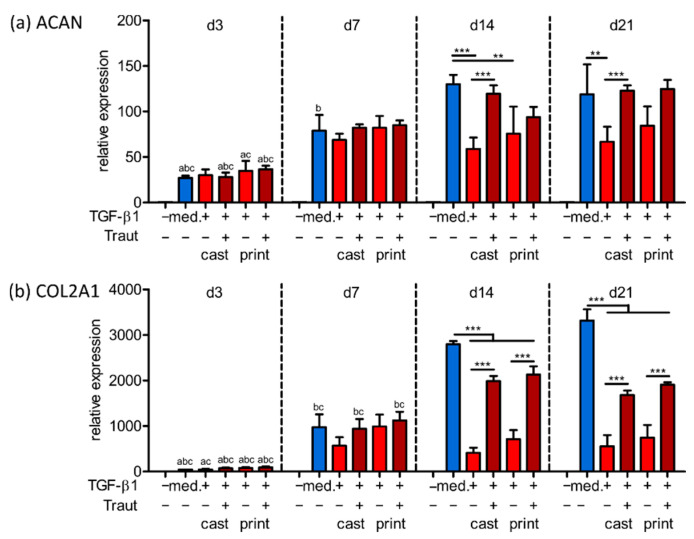
Gene expression analysis of cast and printed constructs. qRT-PCR was performed on gene expression of MSCs in cast and printed constructs with 150 nm mixed (−Traut) or tethered (+Traut) TGF-β1. Printed constructs cultured without or with TGF-β1 as medium supplement served as controls. Expression of (**a**) aggrecan (ACAN) and (**b**) collagen type II (COL2A1) was determined after 3, 7, 14 and 21 days and normalized to EEF1A1 and MSCs in 2D on day 0. Data are presented as mean ± standard deviation (*n* = 3). Significant differences between groups are marked with ** (*p* < 0.01) and *** (*p* < 0.001). Legend: (**a**) Significantly different to corresponding value of the same group at day 7 (at least *p* < 0.05), (**b**) significantly different to corresponding value of the same group at day 14 (at least *p* < 0.05), (**c**) significantly different to corresponding value of the same group at day 21 (at least *p* < 0.05).

As a further step in the signaling cascade, expression of the chondrogenic genes aggrecan and collagen type II, main components of natural cartilage ECM, was evaluated. Cast and printed constructs with 150 nm tethered or mixed TGF-β1 were analyzed after 3, 7, 14 and 21 days and compared to control constructs cultured without or with the protein as medium supplement (Figure 5).

In general, all constructs with MSCs subjected to TGF-β1 showed strongly elevated expression levels compared to MSCs in constructs cultured without TGF-β1. Aggrecan (ACAN) and collagen type II (COL2A1) expression during the first week of culture revealed no significant differences between the TGF-β1 conditions, however, after 14 and 21 days, constructs with tethered TGF-β1 exhibited increased ACAN and COL2A1 expression, as compared to the constructs with mixed TGF-β1 (Figure 5a,b). Furthermore, no impact of the printing process was observed for both genes in all conditions.

#### 2.4.2. ECM Production and Distribution

**Figure 6:**
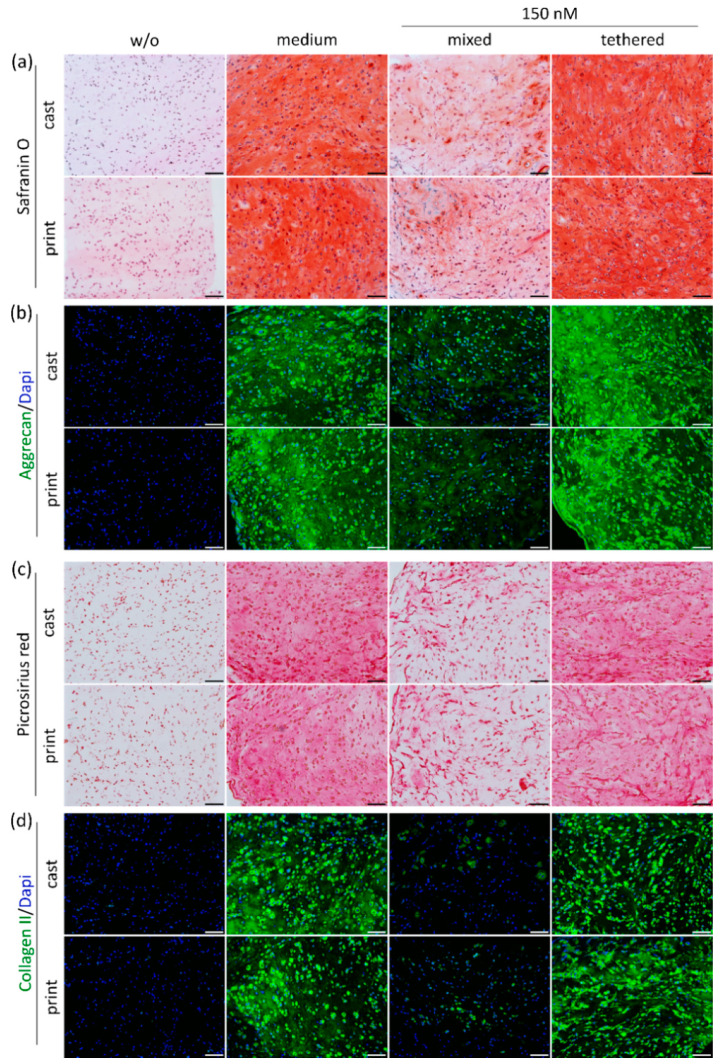
Histological and immunohistochemical (IHC) staining of MSC-derived ECM components in cast and printed constructs. Cast and printed constructs with 150 nm mixed (−Traut) or tethered (+Traut) TGF-β1 were analyzed after 21 days. Constructs cultured without (w/o) or with TGF-β1 as medium supplement (medium) served as controls. GAG production and distribution is depicted by (**a**) safranin O staining and (**b**) IHC staining of aggrecan. Collagen production and distribution is visualized by (**c**) picrosirius red staining and (**d**) IHC staining of collagen type II. Nuclei were counterstained with DAPI. Scale bars represent 100 µm.

An important feature of bioinks for cartilage engineering is the support of MSC differentiation and the necessary porosity for ECM distribution. Figure 6 shows histological and immunohistochemical stainings of cartilage-specific ECM components produced by embedded MSCs during 21 days. Control images of day 1 are presented in the Supplementary Materials (Figure S5).

MSCs cultured in constructs without TGF-β1 were not able to differentiate and produced hardly any ECM components, while constructs in medium supplemented with TGF-β1 showed marked differentiation (Figure 6, left). A concentration of150 nm tethered TGF-β1 yielded strong ECM production and a homogeneous distribution comparable to the medium control, while the same protein concentration without covalent binding (mixed) was distinctly less effective (Figure 6, right). This observation was valid for all stained ECM components, i.e., total GAG and collagen stained by safranin O and picrosirius red (Figure 6a,c), and specific IHC staining of aggrecan and collagen type II (Figure 6b,d). The fibrocartilage marker collagen type I was produced to a low extent in all groups receiving TGF-β1 (Supplementary Materials, Figure S6). Importantly, no difference between cast and printed constructs were determined in any condition.

**Figure 7:**
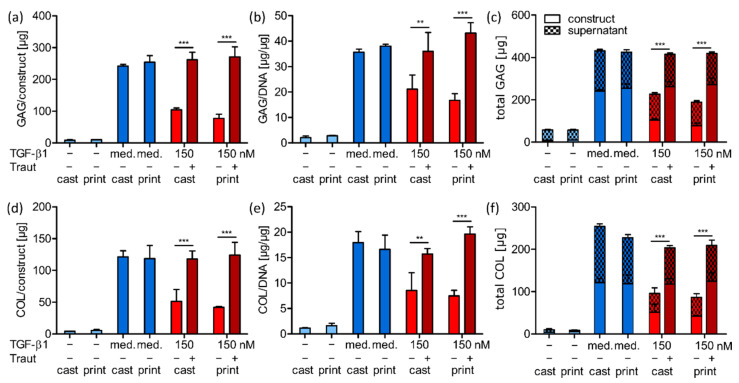
Quantification of MSC-derived ECM components in cast and printed constructs. Cast and printed constructs with 150 nm mixed (−Traut) or tethered (+Traut) TGF-β1 were analyzed after 21 days. Constructs cultured without (−) or with TGF-β1 as medium supplement (med.) served as controls. GAG content is shown for (**a**) the constructs and (**b**) normalized to DNA. (**c**) Total GAG production was quantified by adding the content of the constructs and the collective culture supernatant. Collagen content is shown for (**d**) the constructs and (**e**) normalized to DNA. (**f**) Total collagen production was quantified by adding the content of the constructs and the collective culture supernatant. Data are represented as mean ± standard deviation (*n* = 3). Significant differences are marked with ** (*p* < 0.01) and *** (*p* < 0.001).

To further validate the visual observations, we quantified GAG and collagen contents of all cast and printed constructs after 21 days with biochemical assays (Figure 7). The analysis revealed very low GAG and collagen deposition in constructs cultured without TGF-β1, while constructs with continuous TGF-β1 medium supplementation produced high ECM amounts (Figure 7a,d, blue bars). GAG and collagen contents in constructs with 150 nm tethered TGF-β1 were comparable to the positive control (medium), although no additional growth factor was added during the three weeks of cell culture. MSCs in constructs with 150 nm mixed TGF-β1 produced significantly lower amounts of ECM components compared to constructs in which the same protein concentration was covalently bound (Figure 7a,d, red bars). The same significant differences were detected when GAG and collagen contents were normalized to the DNA content (Figure 7b,e) as well as when total production was compared, i.e., the combined ECM contents in the construct and the supernatant collected during culture time (Figure 7c,f). All initial values at day 1 as well as the analyzed DNA contents can be found in the Supplementary Materials (Figure S7). In general, the quantitative results well confirmed the histological findings and again, there was no difference detected between cast and printed approaches.

#### 2.4.3. Correlation of ECM Production and Construct Stiffness

**Figure 8:**
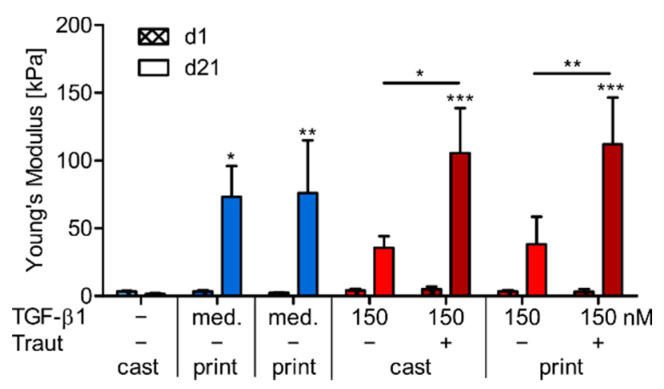
Mechanical characterization of cast and printed constructs. Young’s modulus of cast and printed constructs with 150 nm mixed (−Traut) or tethered (+Traut) TGF-β1 after 1 and 21 days. Constructs cultured without (−) or with TGF-β1 as medium supplement (med.) served as controls. Data are presented as mean ± standard deviation (*n* = 6). Significant differences between groups are marked with * (*p* < 0.05), ** (*p* < 0.01) and *** (*p* < 0.001). Stars above bars on day 21 indicate significant differences to the corresponding value of the same group on day 1.

As an important mechanical feature of engineered cartilage, we analyzed the Young’s modulus of cast and printed constructs with 150 nm tethered or mixed TGF-β1 after construct preparation at day 1 as well as after 21 days of chondrogenic differentiation. Constructs cultured without TGF-β1 or with the soluble protein as continuous medium supplementation served as controls (Figure 8). Initially, all constructs appeared very weak and without significant differences between the conditions (all around 2.5–5.0 kPa at day 1). Constructs cultured without TGF-β1 maintained their weak appearance or even decreased in stiffness within 21 days to around 1.6 kPa. MSCs in constructs with TGF-β1 addition in the culture medium differentiated well and produced high amounts of ECM. This correlated with a significant increase in Young’s modulus to 73.2 kPa for cast and 76.0 kPa for printed constructs (Figure 8, blue bars). Covalently tethered TGF-β1 also induced a significant increase in stiffness to 105.5 kPa for cast and 112.1 kPa for printed constructs after 21 days, while the mixed condition only resulted in 35.6 kPa and 38.2 kPa, respectively (Figure 8, red bars).

In general, bioinks with high polymer contents result in initially stiffer hydrogel networks with good 3D printability, but are often associated with limited cell growth, differentiation and ECM distribution [43]. In a previous study of our group, we demonstrated that the distribution of newly produced ECM molecules was significantly improved in bioinks with a low polymer content which was associated with an increased construct stiffness after chondrogenic differentiation [38]. This was shown for bioinks with 3% polymer content which needed PCL-support structures for 3D printability. The dual-stage crosslinked bioink presented here is stand-alone 3D printable and still allows for an even lower polymer content of 1.4%. Although it exhibited a comparably weak network in the beginning, this bioink enabled homogenous ECM distribution and thereby significantly increased construct stiffness after three weeks in constructs with strong MSC differentiation (tethered TGF-β1 and medium control). Compared to constructs with non-covalently incorporated TGF-β1, MSCs in constructs with tethered TGF-β1 expressed distinctly higher amounts of cartilaginous ECM with a more homogeneous distribution throughout the constructs (Figure 6 and Figure 7), which correlated well with a markedly higher construct stiffness (Figure 8). This was again independent of bioink processing, i.e., no differences between cast and printed constructs were observed.

In conclusion, we established a dual-stage crosslinked hyaluronic acid-based bioink which is 3D printable and enables covalent tethering of TGF-β1. In previous reports, other printable materials were also described for growth factor administration in chondrogenic or osteogenic regeneration approaches, however, proteins were only mixed within the bioinks or administered via nanospheres but not covalently tethered [53,54,55,56,57]. In our study, it was shown that both processes, tethering and 3D printing, did not affect protein functionality or bioink properties. Covalent TGF-β1 tethering enabled prolonged availability for embedded MSCs and markedly enhanced chondrogenic differentiation, as compared to the growth factor non-covalently incorporated in the ink. Furthermore, the low polymer content of the presented ink composition facilitated a penetrable network with chondro-supportive properties and allowed for homogenous ECM distribution and a distinct increase of construct stiffness during chondrogenic differentiation of embedded MSCs. Taken together, this ink composition enabled the generation of high-quality cartilaginous tissues without the need for continuous exogenous growth factor supply and, thus, bears great potential for future investigation towards cartilage regeneration. Moreover, the observation that a tethered growth factor within a printed bioink can lead to superior tissue development may also be explored in other applications in biofabrication.

## 3. Materials and Methods

### 3.1. Materials

All chemicals were purchased from Sigma Aldrich (St. Louis, MO, USA) if not stated differently. Acryloyl chloride stabilized with phenothiazine (abcr GmbH, Karlsruhe, Germany), 1-(3-dimethylaminopropyl)-3-ethylcarbodiimide HCl (EDC, Biosynth CarboSynth, Compton, UK), 1,4-dithiothreitol (DTT, Biosynth CarboSynth, Compton, UK), 2-hydroxy-1-[4-(hydroxyethoxy)-phenyl]-2-methyl-1-propanone (I2959; BASF, Ludwigshafen, Germany), 4-(dimethylamino)benzaldehyde (DAB; Carl Roth, Karlsruhe, Germany), basic fibroblast growth factor (bFGF; BioLegend, London, UK), chloroform-d^1^ (Eurisotope, St-Aubin Cedex, France), DAPI mounting medium ImmunoSelect^®^ (Dako, Hamburg, Germany), deuterium oxide (Deutero GmbH, Kastellaun, Germany), regenerated cellulose dialysis tubes MWCO 3500 Da (Carl Roth, Karlsruhe, Germany), diethylether (Chemobar University of Würzburg, Würzburg, Germany), dimethyl-3,3′-dithiodipropionate (TCI Chemical Industry Co. Ltd., Tokyo, Japan), di-potassium hydrogen phosphate (Merck KGaA, Darmstadt, Germany), di-sodium hydrogen phosphate (Merck KGaA, Darmstadt, Germany), Dulbecco’s Modified Eagle’s Medium high glucose 4.5 g L^−1^ (DMEM; Thermo Scientific, Waltham, MA, USA), Dulbecco’s Modified Eagle’s Medium/Ham’s F-12 (DMEM/F12; Thermo Scientific, Waltham, MA, USA), ethanol (99%, TH Geyer, Renningen, Germany), fetal calf serum (FCS; Thermo Scientific, Waltham, MA, USA), formaldehyde (37%, Carl Roth, Karlsruhe, Germany), hyaluronic acid sodium salt (*M*_W_ 1–2 MDa; Biosynth CarboSynth, Compton, UK), hydrochloric acid (HCl; 32%, 37%, Merck KGaA, Darmstadt, Germany), isopropanol (VWR, Radnor, PA, USA), ITS+ premix (Corning, New York, NY, USA), L-hydroxyprolin (Merck KGaA, Darmstadt, Germany), live/dead cell staining kit (PromoKine, Heidelberg, Germany), methanol (Fisher Scientific, Schwerte, Germany), *N*-hydroxysuccinimide (NHS, Biosynth CarboSynth, Compton, UK), papain (Worthington, Lakewood, CA, USA), penicillin-streptomycin (PS; 100 U mL^−1^ penicillin, 0.1 mg mL^−1^ streptomycin; Thermo Scientific, Waltham, MA, USA), perchloric acid (60%, Merck KGaA, Darmstadt, Germany), phosphate-buffered saline (PBS; Life Technologies, Carlsbad, CA, USA), polyethylene glycol (2-arm PEG, 6 kDa: Sigma Aldrich, St. Louis, MO, USA; 8-arm PEG, 10 kDa: JenKem^®^ Technologies USA, Plano, TX, USA), potassium dihydrogen phosphate (Merck KGaA, Darmstadt, Germany), Proteinase K (Digest-All 4, Life Technologies, Carlsbad, CA, USA), sodium hydrogen carbonate (Merck KGaA, Darmstadt, Germany), sodium hydroxide (Merck KGaA, Darmstadt, Germany), Tissue Tek^®^ O.C.T. (Sakura Finetek, Tokyo, Japan), toluene (Fisher Scientific, Schwerte, Germany), transforming growth factor β1 (TGF-β1; Novoprotein Inc., CA59, Summit, NJ, USA, purchased from PELOBIOTECH GmbH, Munich, Germany), tris(carboxyethyl)phosphine HCl (TCEP, Biosynth CarboSynth, Compton, UK), trypsin-EDTA (0.25%, Life Technologies, Carlsbad, CA, USA).

### 3.2. Synthesis of the Different Bioink Components

The synthesis of the different components of the previously established hyaluronic acid-based bioink (3,3′-dithiobis(propanoic dihydrazide) (DTPH), thiolated hyaluronic acid (HA-SH), polyethylene glycol-diamine, and polyethylene glycol-diallyl carbamate (2-arm PEG-allyl)) was performed as previously published [16]. Synthetical details are given in the Supplementary Materials.

### 3.3. Synthesis of Polyethylene Glycol Octaacrylate (8-Arm PEG-Acryl)

The synthesis of the branched PEG-acrylate necessary for growth factor immobilization was performed using enzyme catalyzed transesterification [58,59]. In brief, 8-arm PEG (5.0 g, 1.0 eq.) was dried in vacuo at 100 °C for 1 h, cooled to 60 °C under argon atmosphere and dissolved in dry toluene (50 mL). Vinyl acrylate (3.0 eq.) and Novozyme 435 (50.0 mg) were added, the suspension was incubated for 3 days at 50 °C and subsequently filtered. After precipitation and washing with diethyl ether, the product was dried in vacuo to form a bulky white solid.

### 3.4. NMR Analysis

^1^H-NMR measurements were performed at a 300 MHz Bruker Biospin spectrometer (Bruker, Billerica, MA) using CDCl_3_ (PEG), d^6^-DMSO (DTP) and D_2_O (HA-SH) as solvents. The solvent peak was set to δ = 7.26 ppm for CDCl_3_, 2.50 ppm for DMSO and 4.79 ppm for D_2_O to which all chemical shifts refer. The degree of substitution of HA-SH was determined by the ratio of the integrals of the thiol carrying substituent and the acetyl amide signal in combination with the anomeric protons of the saccharide backbone. The quantitative PEG-modification was verified by the absence of alterations in the spectra after the addition of trifluoroacetic anhydride to the NMR sample solution.

### 3.5. GPC Analysis

A GPC system from Malvern (Herrenberg, Germany) with a triple detection containing a refractive index detector (VE 3580), a viscometer (270 dual detector) and a multi angle light scattering detector (SEC-MALS 20) was used for GPC analysis. Depending on the molecular weight, different column sets (Malvern, Herrenberg, Germany) were used. For HA-SH samples, two A6000M mixed-bed columns, and for PEG samples, a set of A2000/A3000 columns were chosen. The eluent was prepared using deionized water containing 8.5 g L^−1^ NaNO_3_ and 0.2 g L^−1^ NaN_3_ and the columns were calibrated with PEG standards (Malvern, Herrenberg, Germany). HA-SH samples were dissolved in deionized water with 0.5 g L^−1^ TCEP over 6 h at rt and PEG samples were dissolved in deionized water over 6 h at rt. The obtained data was processed with OmniSEC 5.12 (Malvern, Herrenberg, Germany).

### 3.6. Ink Preparation, TGF-β1 Tethering and 3D Printing

Final composition of the used ink is: 0.5% HA-SH (465 kDa), 0.5% 8-arm PEG-acryl (10 kDa), 0.4% 2-arm PEG-allyl (6 kDa) and 0.05% Irgacure (I2959); all concentrations are indicated as (*w*/*v*). For ink preparation, HA-SH was dissolved in HEPES buffer (pH 7.6, 154 mm), all other components were dissolved in PBS. Three different experimental conditions were prepared: constructs without TGF-β1, constructs with non-covalently bound TGF-β1 (10 nm, 50 nm, 100 nm and 150 nm) and constructs with covalently tethered TGF-β1 (10 nm, 50 nm, 100 nm and 150 nm). For TGF-β1 tethered hydrogels, Traut’s reagent was used for thiol-modification of the protein (4:1 molar excess of Traut’s reagent, 1 h incubation at rt). Subsequently, different amounts of thiolated TGF-β1 were tethered to 8-arm PEG-acryl via Michael addition (1 h at 37 °C). For control constructs with mixed TGF-β1 (no tethering) the same procedure was performed without the addition of Traut’s reagent. Afterwards, all ink components were mixed with or without MSCs (20 × 10^6^ cells mL^−1^, passage 4), transferred to a 3 cc printing cartridge (Nordson EFD, Westlake, OH, USA) and incubated for 30 min at 37 °C to achieve the necessary viscosity for 3D printing via Michael addition. 3D bioprinting as well as printing simulation was performed with an Inkredible+ 3D bioprinter (Cellink, Boston, MA, USA) at 50 kPa through a 330 µm steel nozzle (Nordson EFD, Westlake, OH, USA). G-codes for 3D printed grids and filament fusion tests were generated with the HeartWare software (Cellink, Boston, MA, USA). The NIH ImageJ Fiji software (version 1.52a) was utilized for strand thickness measurements. The CAD file source of the human femoral condyles was MakerBot Thingiverse (object 5820, created by BME_sundevil). The CAD file was sliced with the Heartware Software and printed with a BioX 3D bioprinter (Cellink, Boston, MA, USA). For the printing simulation, bioink was extruded at the same printing conditions used for 3D printing and collected in a tube, subsequently filled in glass molds (5 mm diameter, 2 mm height, 40 µL volume per construct) and irradiated for 10 min at 365 nm (UVL hand lamp with white light filter, 1 mW cm^−^^2^, A. Hartenstein, Würzburg, Germany). Control constructs were cast and irradiated in the same way. MSC-laden constructs were cultured in a chondrogenic differentiation medium (DMEM high glucose medium supplemented with 1% ITS+ premix, 1% PS, 0.1 × 10^−6^ m dexamethasone, 50 μg mL^−1^ l-ascorbic acid 2-phosphate sesquimagnesium salt hydrate and 40 μg mL^−1^ l-proline) at 37 °C and 5% CO_2_. Only constructs of the “medium” control were cultured in chondrogenic differentiation medium supplemented with 10 ng mL^−1^ TGF-β1. The medium was changed thrice per week.

### 3.7. Rheological Analysis

Rheological properties of the hydrogel formulations were characterized using Anton Paar MCR 702 rheometer (Anton Paar, Austria). A parallel plate geometry with a diameter of 25 mm at a 0.5 mm gap was used. A solvent trap was used to minimize evaporation during the experiment. All the experiments were performed with freshly prepared ink solutions in the dark on a pre-heated plate at 37 °C. Time sweep measurements were performed by setting the oscillatory strain amplitude and angular frequency at 0.3% and 10 rad s^−1^, respectively. The viscoelastic properties of the formed network were characterized by an oscillatory frequency sweep between 100–1 rad s^−1^ at 0.3% strain.

### 3.8. Swelling Analysis

Gel wet weight measurements were performed to analyze the swelling and shrinking behavior of constructs without MSCs. Therefore, constructs with 150 nm tethered TGF-β1 or without the growth factor were prepared as described above and incubated in 1 mL PBS for 21 days. Intermediate weight measurements were performed after 30 min, 1 h, 2 h, 4 h, 6 h, 12 h, 1 d, 2 d, 3 d, 5 d, 7 d, 14 d and 21 d and wet weight deviations were calculated in relation to construct weights directly after preparation (0 h).

### 3.9. TGF-β1 Release Analysis

3D constructs with 150 nm mixed or tethered TGF-β1 were prepared as described above and incubated for 21 days, each in 1 mL 1% BSA in PBS. 5 µL samples were taken at 1, 3, 5, 7, 9, 12, 14, 16, 19 and 21 days and subsequently frozen in liquid nitrogen. Quantification of TGF-β1 concentration was performed with a DuoSet ELISA (DY240-05, R&D Systems, Minneapolis, MN, USA).

### 3.10. MSC Isolation and Expansion

Isolation of human bone-marrow derived mesenchymal stromal cells (MSCs) was performed as described previously [11] after informed consent of all patients and with approval of the local ethics committee of the University of Würzburg (186/18). Cells were expanded in DMEM/F12 supplemented with 10% FCS, 1% PS and 3 ng mL^−1^ bFGF at 37 °C and 5% CO_2_.

### 3.11. Cell Viability Analysis and Quantification

Viability of encapsulated MSCs in 3D printed or cast constructs was performed with a live/dead assay cell staining kit (PromoKine, Heidelberg, Germany). Constructs were harvested on d1 or d21, washed in PBS and incubated in the staining solution (4 µm ethidium homodimer III (EthD-III) and 2 µm calcein acetoxymethylester (calcein-AM) in PBS) for 45 min at rt in the dark. After an additional washing step in PBS, cross sections of the constructs were imaged at a fluorescence microscope (Olympus BX51/DP71, Olympus, Hamburg, Germany). For quantification, high magnification images of three printed constructs (three pictures per construct) were analyzed with the Cell Counter plugin of the NIH Image J Fiji Software (version 1.52a), counting green and red cells.

### 3.12. Histological and Immunohistochemical Analysis

MSC-laden constructs were harvested on d1 and d21 and fixed in 3.7% PBS-buffered formaldehyde over night at 4 °C. TissueTek^®^ O.C.T. (Sakura Finetek, Torrance, LA, USA) was used for embedding and 8 µm cryo-sections were performed at a cryostat (CM 3050S, Leica, Wetzlar, Germany). GAG deposition was visualized with safranin O (counterstain: Weigert’s hematoxylin and fast green), and collagen deposition with picrosirius red (counterstain: Weigert’s hematoxylin) [60,61]. Immunohistochemical stainings were performed as previously described [38]. Used antibodies were: anti-aggrecan, 1:300, 969D4D11, Thermo Scientific, Waltham, MA, USA; anti-collagen I, 1:200, ab34710, Abcam, Cambridge, UK; anti-collagen II, 1:1000, II-4C11, Abnova, Taipei, Taiwan; and goat-anti-mouse Alexa488, 1:400, 111-545-003, Jackson ImmunoResearch, Cambridge, UK); all in 1% BSA in PBS. Cells were counterstained with DAPI during mounting (Dako, Hamburg, Germany).

### 3.13. Biochemical Analysis

MSC-laden constructs were homogenized at 25 Hz for 5 min (TissueLyser II, Quiagen, Hilden, Germany) before digesting the suspension with 3 U mL^−1^ papain for 20 h at 60 °C. Quantification of DNA content was performed with Hoechst 33258 (340 nm and 465 nm) and salmon sperm DNA as standard [62]. Dimethylmethyleneblue (DMMB) was used to quantify sulfated glycosaminoglycans (GAG) with chondroitin sulfate as standard at 525 nm [63]. After hydrolysis with hydrochloric acid at 95 °C for 20 h, hydroxyprolines were visualized with 4-(dimethylamino)benzaldehyde at 550 nm. Collagen content was quantified with l-hydroxyproline as standard [64,65]. For calculation of total GAG and collagen content, culture supernatants of MSC-laden constructs were collected at every medium change and also analyzed with the methods described above.

### 3.14. RNA Isolation and Gene Expression Analysis

3D constructs were homogenized for 5 min at 25 Hz before isolating RNA with TRIzol reagent (Invitrogen, Waltham, MA, USA) according to manufacturer’s instructions. cDNA was synthesized with the High-Capacity cDNA Reverse Transcription Kit (Applied Biosystems, Foster City, CA, USA) and real-time qRT-PCR was performed with the MESA GREEN qPCR Mastermix Plus for SYBR^®^ Assay (Eurogentec, Liège, Belgium) in a CFX96 real-time system (Bio-Rad, Hercules, CA, USA). Relative gene expression of all samples was normalized to the eukaryotic translation initiation factor 1α1 (EEF1A1) and to gene expression of MSC 2D samples on d0, all analyzed with the 2^−∆∆CT^ method [66]. A table with self-designed primer sequences can be found in the Supplementary Materials (Table S1).

### 3.15. Protein Separation and Western Blot Analysis

3D constructs were homogenized for 5 min at 25 Hz in RIPA lysis and extraction buffer (Thermo Scientific, Waltham, MA, USA) supplemented with Pierce protease and phosphatase inhibitor (Thermo Scientific, Waltham, MA, USA). Protein isolation was performed by repeated freeze-thaw cycles before protein concentration was analyzed with the Pierce™ BCA protein assay kit (Thermo Scientific, Waltham, MA, USA) according to manufacturer’s instructions. Protein separation was performed at 80 V by tris-glycine SDS-PAGE under reducing conditions using a 10% polyacrylamide gel. For Western blotting, proteins were transferred to a Immobilon-FL PVDF membrane (0.45 µm; Merck KGaA, Darmstadt, Germany) at 100 V for 90 min on ice before blocking with 1 × ROTI^®^ Block (Carl Roth, Karlsruhe, Germany) in tris-buffered saline supplemented with 0.1% tween-20 (TBST) for 1 h at rt. Primary antibodies against Smad2/3 (Cell Signaling, 3102, rabbit, 1:1000), Phospho-Smad3 (Abcam, ab52903, rabbit, 1:1000) and GAPDH (Merck, MAB374, mouse, 1:5000) were diluted in 0.5 × ROTI^®^ Block/TBS and incubated over night at 4 °C under agitation. After washing in TBST, secondary antibodies (Goat-anti-Rabbit 800CW IRDye, 926-32211, 1:15,000, and Goat-anti-Mouse 680CW IRDye, 925-68070, 1:15,000; LI-COR, Bad Homburg, Germany) were incubated for 1 h at rt under agitation in the dark. Subsequently, membranes were washed in TBST before protein detection was performed at a LI-COR Odyssey^®^ Fc imaging system.

### 3.16. Mechanical Analysis

MSC-laden constructs were analyzed with a load cell of 250 g at a dynamical mechanical testing machine (ElectroForce 5500, Bose, Eden Prairie, MN, USA) on d1 and d21. A constant cross head displacement rate of 0.001 mm s^−1^ was used to compress the constructs to a final depth of 0.5 mm. The Young’s modulus was determined as the slope of the true stress-strain curve in the linear elastic range.

### 3.17. Statistical Analysis

Data is represented as mean ± standard deviation of at least three replicates per condition. Significant differences were marked as follows: * (*p* < 0.05), ** (*p* < 0.01) and *** (*p* < 0.001) and were calculated with GraphPad Prism 5 software. For comparison of multiple groups at one time point, a one-way ANOVA with Bonferroni posthoc test was used. A two-way ANOVA with Bonferroni posthoc test was performed for comparison of multiple groups at different time points.

## Data Availability

Data is contained within the article.

## Supplementary Materials

Supplementary materials are available online at www.mdpi.com/article/10.3390/ijms23020924/s1. References [11,67,68] are cited in the Supplementary Materials.

## References

[1] Groll J., Boland T., Blunk T., Burdick J.A., Cho D.W., Dalton P.D., Derby B., Forgacs G., Li Q., Mironov V.A. (2016). Biofabrication: Reappraising the definition of an evolving field. Biofabrication.

[2] Moroni L., Boland T., Burdick J.A., De Maria C., Derby B., Forgacs G., Groll J., Li Q., Malda J., Mironov V.A. (2018). Biofabrication: A Guide to Technology and Terminology. Trends Biotechnol..

[3] Levato R., Jungst T., Scheuring R.G., Blunk T., Groll J., Malda J. (2020). From Shape to Function: The Next Step in Bioprinting. Adv. Mater..

[4] Mouser V.H.M., Levato R., Bonassar L.J., D’Lima D.D., Grande D.A., Klein T.J., Saris D.B.F., Zenobi-Wong M., Gawlitta D., Malda J. (2017). Three-Dimensional Bioprinting and Its Potential in the Field of Articular Cartilage Regeneration. Cartilage.

[5] Wu Y., Kennedy P., Bonazza N., Yu Y., Dhawan A., Ozbolat I. (2021). Three-Dimensional Bioprinting of Articular Cartilage: A Systematic Review. Cartilage.

[6] Moroni L., Burdick J.A., Highley C., Lee S.J., Morimoto Y., Takeuchi S., Yoo J.J. (2018). Biofabrication strategies for 3D in vitro models and regenerative medicine. Nat. Rev. Mater..

[7] Onofrillo C., Duchi S., O’Connell C.D., Blanchard R., O’Connor A.J., Scott M., Wallace G.G., Choong P.F.M., Di Bella C. (2018). Biofabrication of human articular cartilage: A path towards the development of a clinical treatment. Biofabrication.

[8] Petta D., D’Amora U., Ambrosio L., Grijpma D.W., Eglin D., D’Este M. (2020). Hyaluronic acid as a bioink for extrusion-based 3D printing. Biofabrication.

[9] Burdick J.A., Prestwich G.D. (2011). Hyaluronic acid hydrogels for biomedical applications. Adv. Mater..

[10] Highley C.B., Prestwich G.D., Burdick J.A. (2016). Recent advances in hyaluronic acid hydrogels for biomedical applications. Curr. Opin. Biotechnol..

[11] Stichler S., Böck T., Paxton N., Bertlein S., Levato R., Schill V., Smolan W., Malda J., Teßmar J., Blunk T. (2017). Double printing of hyaluronic acid/poly(glycidol) hybrid hydrogels with poly(ε-caprolactone) for MSC chondrogenesis. Biofabrication.

[12] Poldervaart M.T., Goversen B., de Ruijter M., Abbadessa A., Melchels F.P.W., Öner F.C., Dhert W.J.A., Vermonden T., Alblas J. (2017). 3D bioprinting of methacrylated hyaluronic acid (MeHA) hydrogel with intrinsic osteogenicity. PLoS ONE.

[13] Pan H.M., Chen S., Jang T.-S., Han W.T., Jung H.-D., Li Y., Song J. (2019). Plant seed-inspired cell protection, dormancy, and growth for large-scale biofabrication. Biofabrication.

[14] Petta D., Grijpma D.W., Alini M., Eglin D., D’Este M. (2018). Three-Dimensional Printing of a Tyramine Hyaluronan Derivative with Double Gelation Mechanism for Independent Tuning of Shear Thinning and Postprinting Curing. ACS Biomater. Sci. Eng..

[15] Galarraga J.H., Locke R.C., Witherel C.E., Stoeckl B.D., Castilho M., Mauck R.L., Malda J., Levato R., Burdick J.A. (2021). Fabrication of MSC-laden composites of hyaluronic acid hydrogels reinforced with MEW scaffolds for cartilage repair. Biofabrication.

[16] Hauptstein J., Forster L., Nadernezhad A., Horder H., Stahlhut P., Groll J., Blunk T., Teßmar J. (2021). Bioink Platform Utilizing Dual-stage Crosslinking of Hyaluronic Acid Tailored for Chondrogenic Differentiation of Mesenchymal Stromal Cells. Macromol. Biosci..

[17] Redini F., Daireaux M., Mauviel A., Galera P., Loyau G., Pujol J.P. (1991). Characterization of proteoglycans synthesized by rabbit articular chondrocytes in response to transforming growth factor-beta (TGF-beta). Biochim. Biophys. Acta.

[18] Blaney Davidson E.N., Vitters E.L., van der Kraan P.M., van den Berg W.B. (2006). Expression of transforming growth factor-beta (TGFbeta) and the TGFbeta signalling molecule SMAD-2P in spontaneous and instability-induced osteoarthritis: Role in cartilage degradation, chondrogenesis and osteophyte formation. Ann. Rheum. Dis..

[19] Lee S.J. (2000). Cytokine delivery and tissue engineering. Yonsei Med. J..

[20] van Beuningen H.M., van der Kraan P.M., Arntz O.J., van den Berg W.B. (1994). Transforming growth factor-beta 1 stimulates articular chondrocyte proteoglycan synthesis and induces osteophyte formation in the murine knee joint. Lab. Invest..

[21] Blaney Davidson E.N., van der Kraan P.M., van den Berg W.B. (2007). TGF-beta and osteoarthritis. Osteoarthr. Cartil..

[22] Madry H., Rey-Rico A., Venkatesan J.K., Johnstone B., Cucchiarini M. (2014). Transforming growth factor Beta-releasing scaffolds for cartilage tissue engineering. Tissue Eng. Part B.

[23] Zhu J., Marchant R.E. (2011). Design properties of hydrogel tissue-engineering scaffolds. Expert Rev. Med. Devices.

[24] Böck T., Schill V., Krähnke M., Steinert A.F., Tessmar J., Blunk T., Groll J. (2018). TGF-β1-Modified Hyaluronic Acid/Poly(glycidol) Hydrogels for Chondrogenic Differentiation of Human Mesenchymal Stromal Cells. Macromol. Biosci..

[25] Sridhar B.V., Doyle N.R., Randolph M.A., Anseth K.S. (2014). Covalently tethered TGF-β1 with encapsulated chondrocytes in a PEG hydrogel system enhances extracellular matrix production. J. Biomed. Mater. Res. Part A.

[26] McCall J.D., Luoma J.E., Anseth K.S. (2012). Covalently tethered transforming growth factor beta in PEG hydrogels promotes chondrogenic differentiation of encapsulated human mesenchymal stem cells. Drug Deliv. Transl. Res..

[27] Blaeser A., Duarte Campos D.F., Puster U., Richtering W., Stevens M.M., Fischer H. (2016). Controlling Shear Stress in 3D Bioprinting is a Key Factor to Balance Printing Resolution and Stem Cell Integrity. Adv. Healthcare Mater..

[28] Lepowsky E., Muradoglu M., Tasoglu S. (2018). Towards preserving post-printing cell viability and improving the resolution: Past, present, and future of 3D bioprinting theory. Bioprinting.

[29] Blaeser A., Duarte Campos D.F., Puster U., Fischer H. (2015). Shear Stress in 3D-Bioprinting strongly impacts Human MSC Survival and Proliferation Potential. BioNanoMaterials.

[30] Müller S.J., Mirzahossein E., Iftekhar E.N., Bächer C., Schrüfer S., Schubert D.W., Fabry B., Gekle S. (2020). Flow and hydrodynamic shear stress inside a printing needle during biofabrication. PLoS ONE.

[31] Bekard I.B., Asimakis P., Bertolini J., Dunstan D.E. (2011). The effects of shear flow on protein structure and function. Biopolymers.

[32] Thomas C.R., Geer D. (2011). Effects of shear on proteins in solution. Biotechnol. Lett..

[33] Schneider M.C., Chu S., Randolph M.A., Bryant S.J. (2019). An in vitro and in vivo comparison of cartilage growth in chondrocyte-laden matrix metalloproteinase-sensitive poly(ethylene glycol) hydrogels with localized transforming growth factor β3. Acta Biomater..

[34] Kopesky P.W., Vanderploeg E.J., Kisiday J.D., Frisbie D.D., Sandy J.D., Grodzinsky A.J. (2011). Controlled delivery of transforming growth factor β1 by self-assembling peptide hydrogels induces chondrogenesis of bone marrow stromal cells and modulates Smad2/3 signaling. Tissue Eng. Part A.

[35] Choi B., Kim S., Fan J., Kowalski T., Petrigliano F., Evseenko D., Lee M. (2015). Covalently conjugated transforming growth factor-β1 in modular chitosan hydrogels for the effective treatment of articular cartilage defects. Biomater. Sci..

[36] Erickson I.E., Huang A.H., Sengupta S., Kestle S., Burdick J.A., Mauck R.L. (2009). Macromer density influences mesenchymal stem cell chondrogenesis and maturation in photocrosslinked hyaluronic acid hydrogels. Osteoarthr. Cartil..

[37] Bian L., Hou C., Tous E., Rai R., Mauck R.L., Burdick J.A. (2013). The influence of hyaluronic acid hydrogel crosslinking density and macromolecular diffusivity on human MSC chondrogenesis and hypertrophy. Biomaterials.

[38] Hauptstein J., Böck T., Bartolf-Kopp M., Forster L., Stahlhut P., Nadernezhad A., Blahetek G., Zernecke-Madsen A., Detsch R., Jüngst T. (2020). Hyaluronic Acid-Based Bioink Composition Enabling 3D Bioprinting and Improving Quality of Deposited Cartilaginous Extracellular Matrix. Adv. Healthc. Mater..

[39] Deng Y., Sun A.X., Overholt K.J., Yu G.Z., Fritch M.R., Alexander P.G., Shen H., Tuan R.S., Lin H. (2019). Enhancing chondrogenesis and mechanical strength retention in physiologically relevant hydrogels with incorporation of hyaluronic acid and direct loading of TGF-β. Acta Biomater..

[40] Lam J., Truong N.F., Segura T. (2014). Design of cell-matrix interactions in hyaluronic acid hydrogel scaffolds. Acta Biomater..

[41] Kim H.D., Valentini R.F. (2002). Retention and activity of BMP-2 in hyaluronic acid-based scaffolds in vitro. J. Biomed. Mater. Res..

[42] Cai S., Liu Y., Zheng Shu X., Prestwich G.D. (2005). Injectable glycosaminoglycan hydrogels for controlled release of human basic fibroblast growth factor. Biomaterials.

[43] Malda J., Visser J., Melchels F.P., Jüngst T., Hennink W.E., Dhert W.J.A., Groll J., Hutmacher D.W. (2013). 25th Anniversary Article: Engineering Hydrogels for Biofabrication. Adv. Mater..

[44] Tzavlaki K., Moustakas A. (2020). TGF-β Signaling. Biomolecules.

[45] Huang F., Chen Y.-G. (2012). Regulation of TGF-β receptor activity. Cell Biosci..

[46] Lucas L., Aravind A., Emma P., Christophe M., Edwin-Joffrey C. (2021). Rheology, simulation and data analysis toward bioprinting cell viability awareness. Bioprinting.

[47] Askari M., Naniz M.A., Kouhi M., Saberi A., Zolfagharian A., Bodaghi M. (2020). Recent progress in extrusion 3D bioprinting of hydrogel biomaterials for tissue regeneration: A comprehensive review with focus on advanced fabrication techniques. Biomater. Sci..

[48] McMurtrey R.J. (2016). Analytic Models of Oxygen and Nutrient Diffusion, Metabolism Dynamics, and Architecture Optimization in Three-Dimensional Tissue Constructs with Applications and Insights in Cerebral Organoids. Tissue Eng. Part C.

[49] McMurtrey R.J. (2017). Roles of Diffusion Dynamics in Stem Cell Signaling and Three-Dimensional Tissue Development. Stem Cells Dev..

[50] Weiss A., Attisano L. (2013). The TGFbeta superfamily signaling pathway. Wiley Interdiscip. Rev. Dev. Biol..

[51] Song B., Estrada K.D., Lyons K.M. (2009). Smad signaling in skeletal development and regeneration. Cytokine Growth Factor Rev..

[52] Wang W., Rigueur D., Lyons K.M. (2014). TGFβ signaling in cartilage development and maintenance. Birth Defects Res. Part C.

[53] Zhang X., Liu Y., Luo C., Zhai C., Li Z., Zhang Y., Yuan T., Dong S., Zhang J., Fan W. (2021). Crosslinker-free silk/decellularized extracellular matrix porous bioink for 3D bioprinting-based cartilage tissue engineering. Mater. Sci. Eng. C.

[54] Zhu W., Cui H., Boualam B., Masood F., Flynn E., Rao R.D., Zhang Z.Y., Zhang L.G. (2018). 3D bioprinting mesenchymal stem cell-laden construct with core-shell nanospheres for cartilage tissue engineering. Nanotechnology.

[55] Wang B., Díaz-Payno P.J., Browe D.C., Freeman F.E., Nulty J., Burdis R., Kelly D.J. (2021). Affinity-bound growth factor within sulfated interpenetrating network bioinks for bioprinting cartilaginous tissues. Acta Biomater..

[56] Du M., Chen B., Meng Q., Liu S., Zheng X., Zhang C., Wang H., Li H., Wang N., Dai J. (2015). 3D bioprinting of BMSC-laden methacrylamide gelatin scaffolds with CBD-BMP2-collagen microfibers. Biofabrication.

[57] Park J.H., Gillispie G.J., Copus J.S., Zhang W., Atala A., Yoo J.J., Yelick P.C., Lee S.J. (2020). The effect of BMP-mimetic peptide tethering bioinks on the differentiation of dental pulp stem cells (DPSCs) in 3D bioprinted dental constructs. Biofabrication.

[58] Warwel S., Steinke G., Klaas M.R.G. (1996). An efficient method for lipase-catalysed preparation of acrylic and methacrylic acid esters. Biotechnol. Tech..

[59] Heeres A., Vanbroekhoven K., Van Hecke W. (2019). Solvent-free lipase-catalyzed production of (meth)acrylate monomers: Experimental results and kinetic modeling. Biochem. Eng. J..

[60] Schmitz N., Laverty S., Kraus V.B., Aigner T. (2010). Basic methods in histopathology of joint tissues. Osteoarthr. Cartil..

[61] Martin I., Obradovic B., Freed L.E., Vunjak-Novakovic G. (1999). Method for quantitative analysis of glycosaminoglycan distribution in cultured natural and engineered cartilage. Ann. Biomed. Eng..

[62] Kim Y.J., Sah R.L., Doong J.Y., Grodzinsky A.J. (1988). Fluorometric assay of DNA in cartilage explants using Hoechst 33258. Anal. Biochem..

[63] Farndale R.W., Buttle D.J., Barrett A.J. (1986). Improved quantitation and discrimination of sulphated glycosaminoglycans by use of dimethylmethylene blue. Biochim. Biophys. Acta.

[64] Woessner J.F. (1961). The determination of hydroxyproline in tissue and protein samples containing small proportions of this imino acid. Arch. Biochem. Biophys..

[65] Hollander A.P., Heathfield T.F., Webber C., Iwata Y., Bourne R., Rorabeck C., Poole A.R. (1994). Increased damage to type II collagen in osteoarthritic articular cartilage detected by a new immunoassay. J. Clin. Investig..

[66] Livak K.J., Schmittgen T.D. (2001). Analysis of relative gene expression data using real-time quantitative PCR and the 2(-Delta Delta C(T)) Method. Methods.

[67] Vercruysse K.P., Marecak D.M., Marecek J.F., Prestwich G.D. (1997). Synthesis and in Vitro Degradation of New Polyvalent Hydrazide Cross-Linked Hydrogels of Hyaluronic Acid. Bioconjugate Chem..

[68] Iijima M., Ulkoski D., Sakuma S., Matsukuma D., Nishiyama N., Otsuka H., Scholz C. (2016). Synthesis of PEGylated poly(amino acid) pentablock copolymers and their self-assembly. Polym. Int..

