# Supplementary material for: Tethered TGF-β1 in a Hyaluronic Acid-Based Bioink for Bioprinting Cartilaginous Tissues
Source: Int J Mol Sci. 2022 Jan 15;23(2):924. doi: 10.3390/ijms23020924 (PMC8781121; doi:10.3390/ijms23020924)
Supplement: Supplementary file 1 [file ijms-23-00924-s001.zip › ijms-1546658-supplementary final.pdf]

Supplementary Materials

Tethered TGF-β1 in a Hyaluronic Acid-Based Bioink for Bioprinting Cartilaginous Tissues

Julia Hauptstein [1], Leonard Forster [2], Ali Nadernezhad [2], Jürgen Groll [2], Jörg Teßmar [2] and Torsten Blunk [1,*]

[1] Department of Trauma, Hand, Plastic and Reconstructive Surgery, University of Würzburg, 97080 Würzburg, Germany; hauptstein_j@ukw.de
[2] Department for Functional Materials in Medicine and Dentistry, Bavarian Polymer Institute (BPI), University of Würzburg, 97070 Würzburg, Germany; leonard.forster@fmz.uni-wuerzburg.de (L.F.); ali.nadernezhad@fmz.uni-wuerzburg.de (A.N.); juergen.groll@fmz.uni-wuerzburg.de (J.G.); joerg.tessmar@fmz.uni-wuerzburg.de (J.T.)
* Correspondence: blunk_t@ukw.de; Tel.: +49-931-201-37115

Figure S1

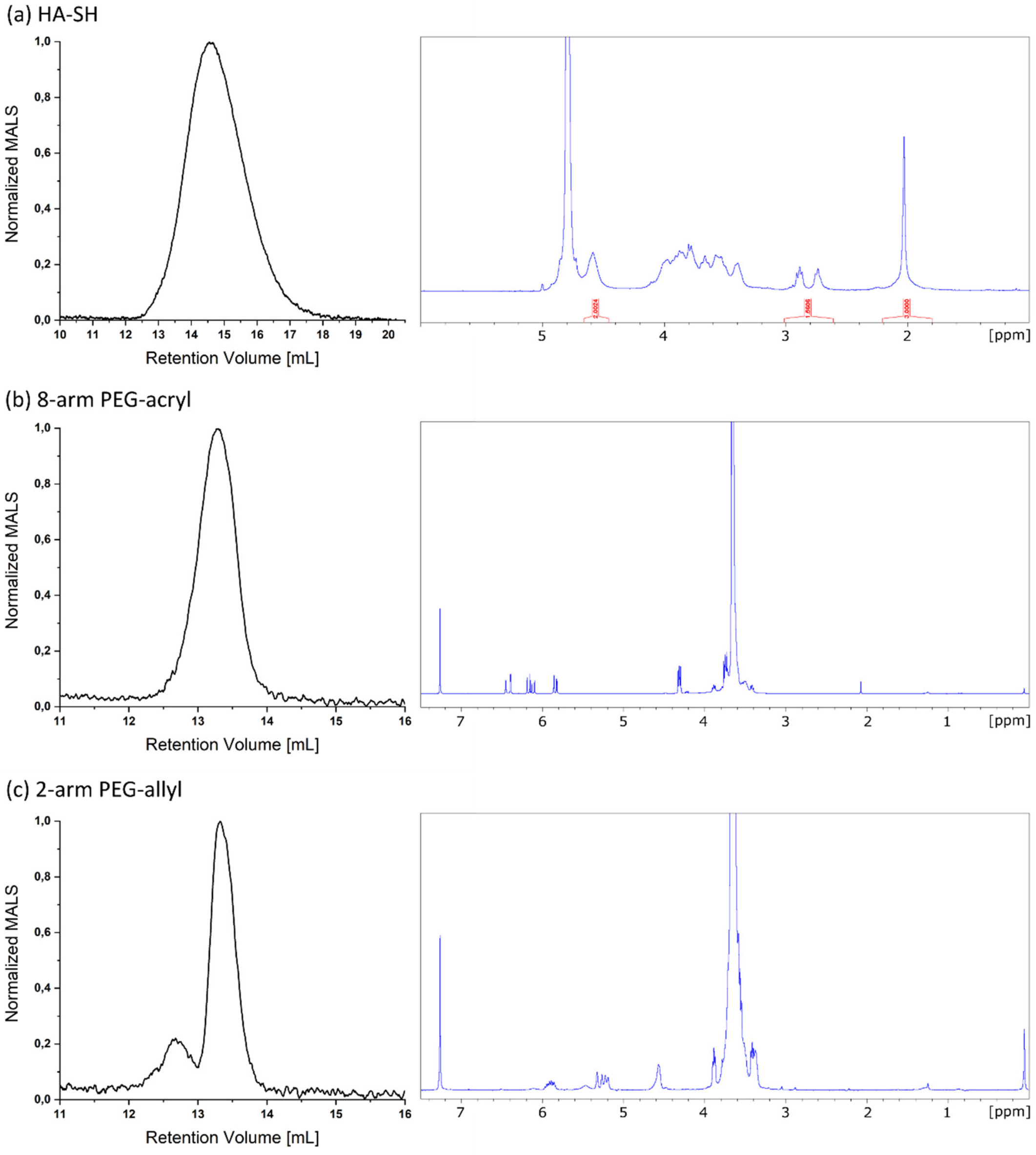

Figure S1: Characterization of bioink components. GPC elugrams (left) and ^{1}H-NMR spectra (right) of (**a**) HA-SH (300-MHz, D_2O) δ (ppm) = 1.82-2.20 (s, 3 H, *N*-acetyl), 2.57-3.00 (m, thiol-modification), 3.20-4.20 (m, 10 H, HA-backbone), 4.40-4.70 (s, 2 H, anomeric protons); (**b**) 8-arm PEG-acryl (300 MHz, $CDCl_3$) δ (ppm) = 3.33-3.99 (m, PEG-backbone and satellites), 4.18-4.38 (t, 2 H, PEG-O-CH_2-CH_2-O-acryl) 5.76-6.50 (d/dd, CH=CH_2) after TFAA addition; and (**c**) 2-arm PEG-allyl (300 MHz, $CDCl_3$) δ (ppm) = 3.15-4.09 (m, PEG-backbone and satellites), 4.43-4.76 (m, PEG-O-CH_2-CH_2-N-), 5.11-6.07 (d/d/m, O-CH_2-CH=CH_2), 5.38 (s, -NH-C(=O)-O-) after TFAA addition.

Figure S2

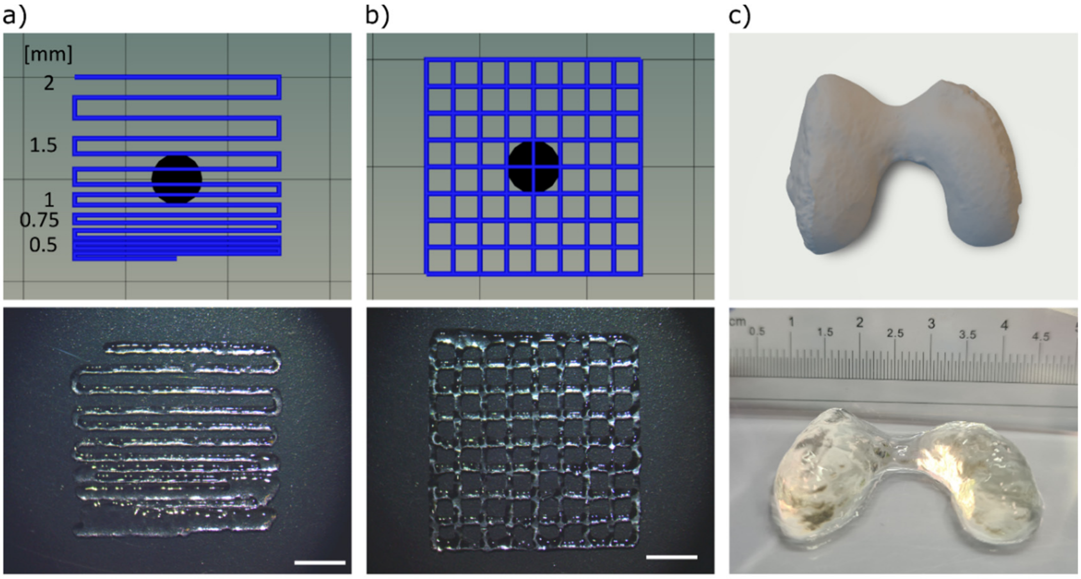

Figure S2: 3D printability assessment. (**a**) Filament fusion test (strand distance indicated next to the G-code) and (**b**) 2-layered grid structure used for strand thickness and intersection diagonal measurements. G-codes are represented in the upper row, printed constructs in the lower row. Scale bars represent 5 mm. (**c**) CAD model of human femoral condyles (top) and the corresponding 3D printed 21-layered construct (bottom).

Figure S3

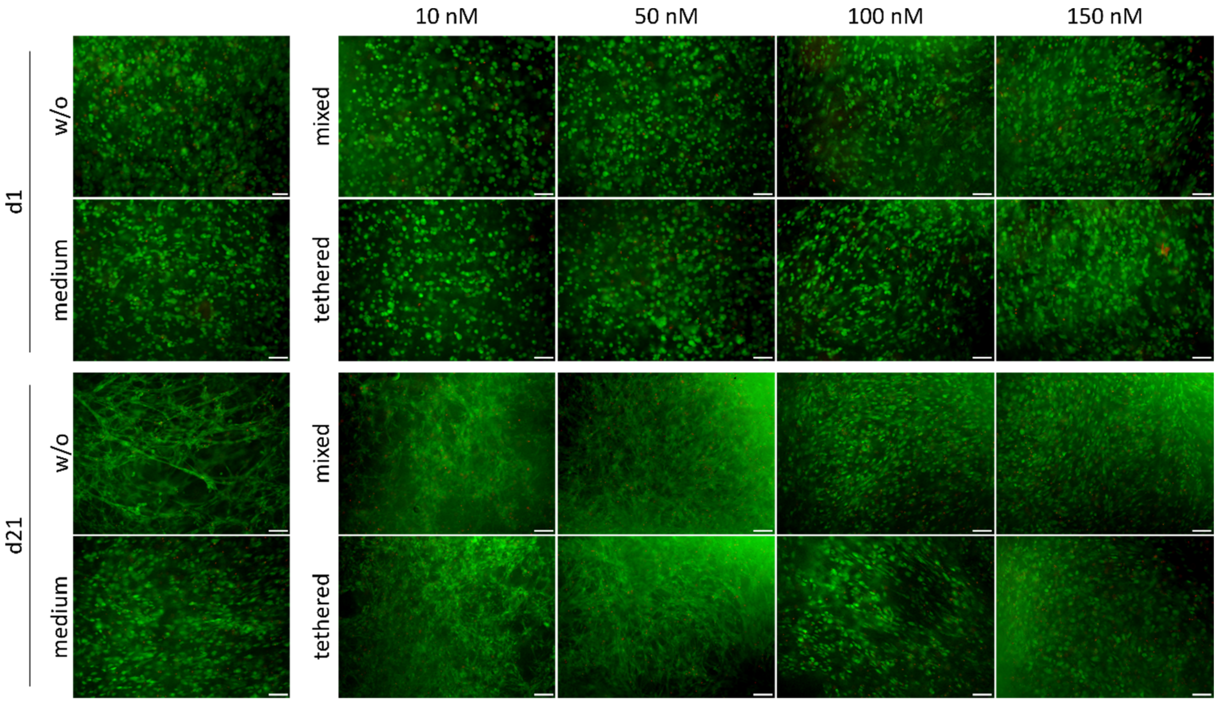

Figure S3: Cell survival of MSCs in cast constructs. Survival was analyzed on day 1 and 21 in constructs with 10, 50, 100 or 150 nM mixed or tethered TGF-β1, as well as control groups cultured without (w/o) or with TGF-β1 as medium supplement (medium). Living cells are stained with calcein-AM and appear in green, dead cells are stained with EthD-III and appear red. Scale bars represent 100 µm.

Figure S4

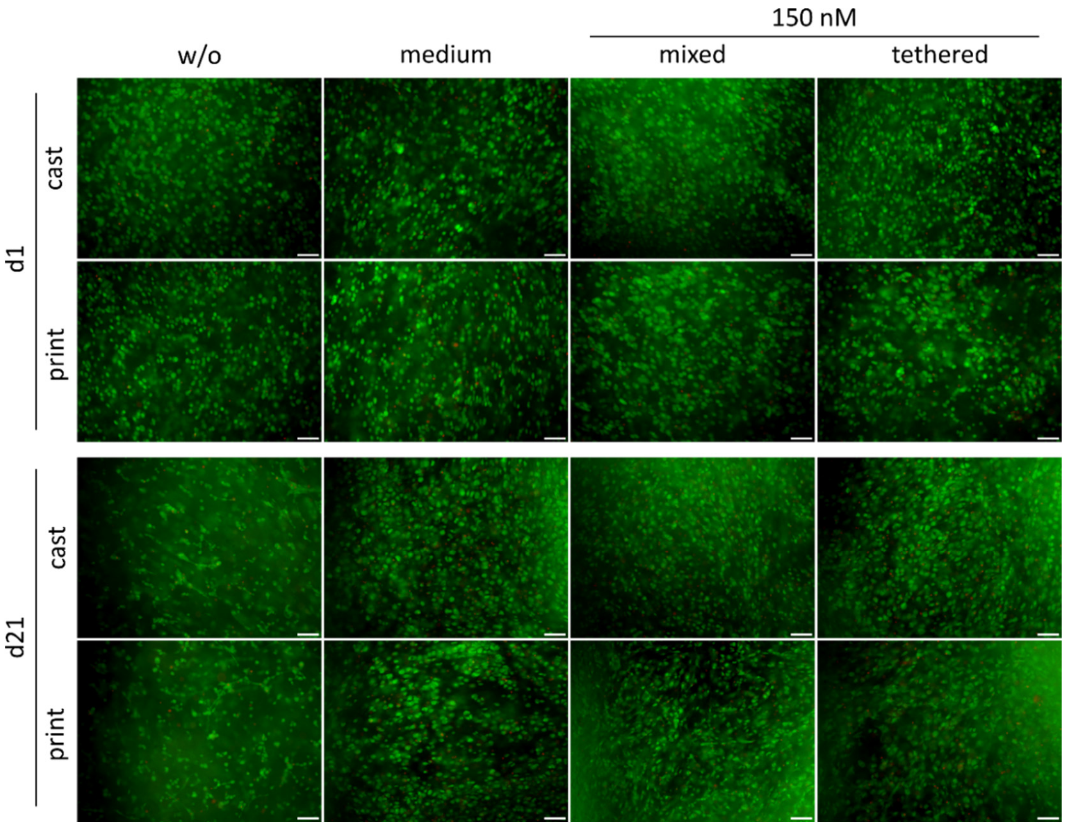

Figure S4: Cell survival of MSCs in cast and printed constructs. Cell survival was analyzed after 1 and 21 days in cast and printed constructs with 150 nM mixed (- Traut) or tethered (+ Traut) TGF-β1. Constructs cultured without (w/o) or with TGF-β1 as medium supplement (medium) served as control. Living cells are stained with calcein-AM and appear in green, dead cells are stained with EthD-III and appear red. Scale bars represent 100 µm.

Figure S5

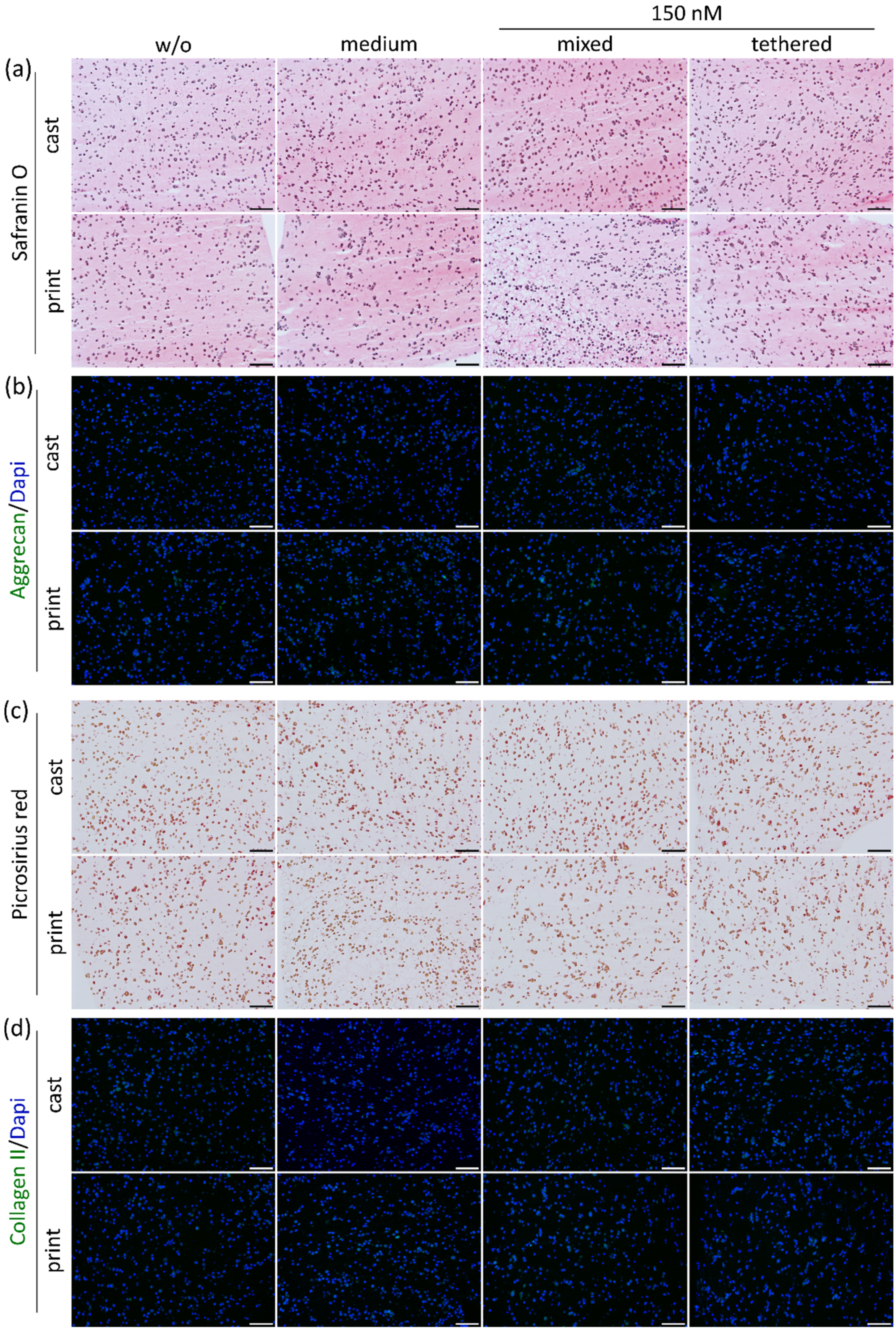

Figure S5: Histological and immunohistochemical (IHC) staining of ECM components in cast and printed constructs after 1 day. Cast and printed constructs were stained for (**a**) GAG (safranin O) (**b**) aggrecan, (**c**) collagen (picrosirius red) and (**d**) collagen type II. Nuclei were counterstained with DAPI. Scale bars represent 100 µm.

Figure S6

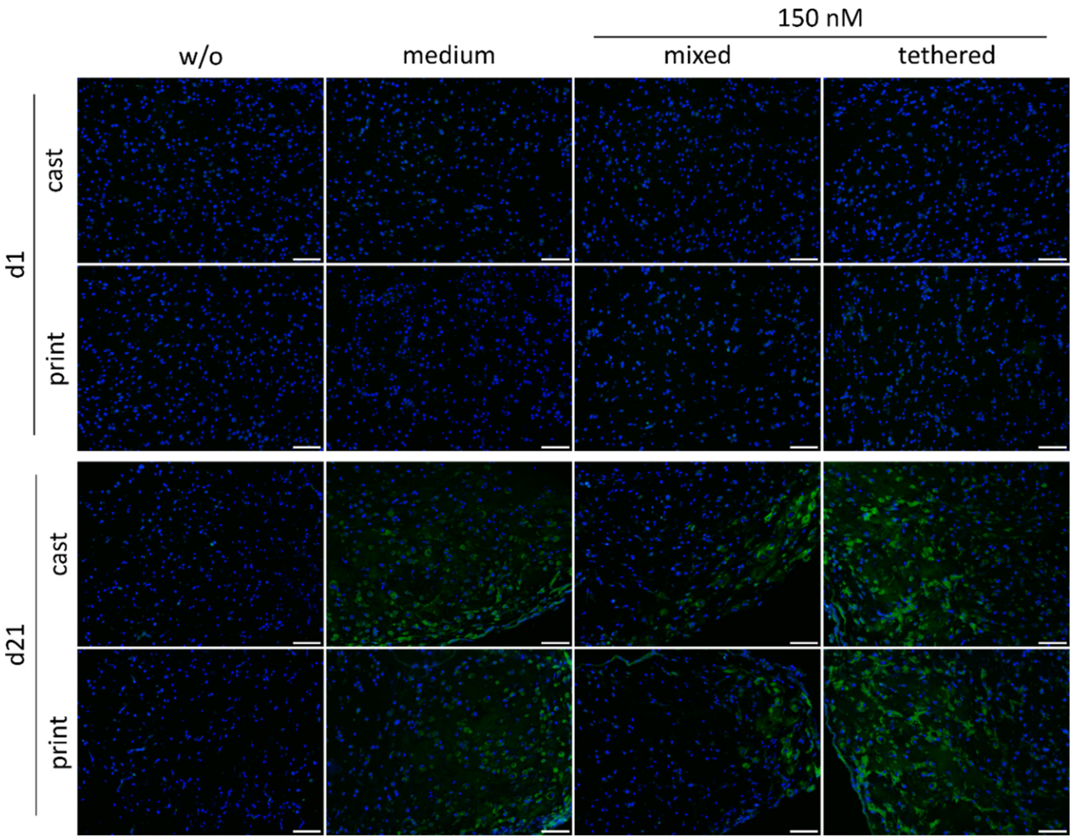

Figure S6: Immunohistochemical (IHC) staining of collagen type I in cast and printed constructs. Collagen type I was stained after 1 and 21 days in cast and printed constructs with 150 nM mixed (- Traut) or tethered (+ Traut) TGF-β1. Constructs cultured without or with TGF-β1 as medium supplement served as control. Nuclei were counterstained with DAPI. Scale bars represent 100 μm.

Figure S7

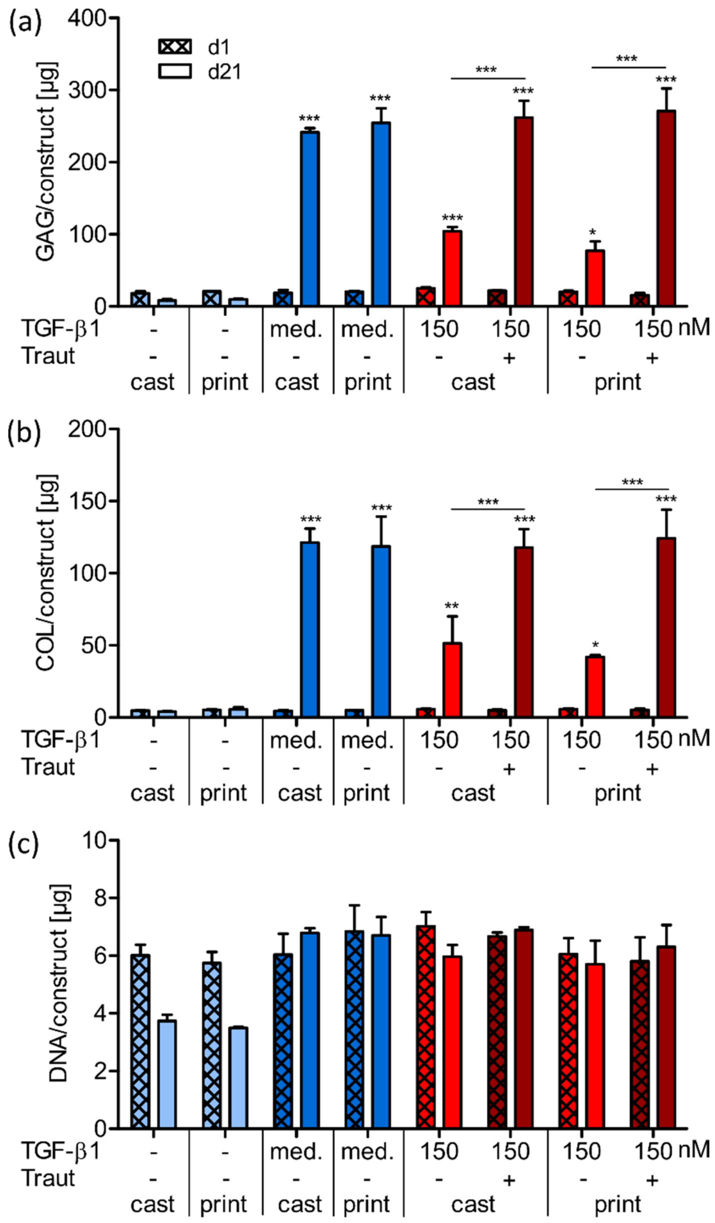

Figure S7: Quantification of ECM components at day 1 and 21 in cast and printed constructs. Quantification of (**a**) GAG, (**b**) collagen and (**c**) DNA content in constructs with 150 nM mixed (- Traut) or tethered (+ Traut) TGF-β1. Constructs cultured without (-) or with TGF-β1 as medium supplement (med.) served as control. Data are represented as mean ± standard deviation ($n = 3$). Significant differences are marked with * ($p < 0.05$), ** ($p < 0.01$) and *** ($p < 0.001$). Stars above bars on day 21 indicate significant differences to the corresponding value of the same group on day 1.

Table S1: qRT-PCR primer sequences.

primer	sequence
ACAN forward	5'- TACGACGCCATCTGCTACAC-3'
ACAN reverse	5'- GACGGTGATGTCCTCCTCAC-3'
COL2A1 forward	5'- CTGTCCTTCGGTGTCAGGG-3'
COL2A1 reverse	5'- CAGACACAGATCCGGCAGG-3'
EEF1A1 forward	5'- GCCCATGTGTGTTGAGAGC-3'
EEF1A1 reverse	5'- CCGCAACTGTCTGTCTCATATC-3'

Synthesis of the different ink components

Synthesis of 3,3′-dithiobis(propanoic dihydrazide) (DTPH): The synthesis was performed according to Vercruysse et al. [2] with slight modifications. In brief, dimethyl 3,3'-dithiodipropionate (1 eq.) was dissolved in methanol, cooled to 0 °C and an excess of hydrazine monohydrate (8.0 eq.) was added. The reaction solution was stirred at 0 °C for 4 h following another 16 h at rt. The supernatant of the resulting suspension was discarded, the sediment was washed with methanol and subsequently with diethyl ether. The final product was dried in vacuo to form a white, shiny solid.

Synthesis of thiolated hyaluronic acid (HA-SH): The synthesis was performed according to Stichler et al. [1] with slight modifications. Hyaluronic acid (HA) (3.0 g, 1.0 eq.) was dissolved in 300 mL MilliQ overnight at 37 °C. NHS (4.0 eq.) and DTPH (4.0 eq.) were dissolved in 300 mL Sørensen buffer (0.2 M, pH 5.5) at rt, both solutions were combined and mixed. EDC (7.5 eq.) was added, the pH was lowered to 4.0 with 10 % HCl and the mixture was incubated for 4 h at 37 °C. After a pH adjustment to over 8.5 with 2 N NaOH, DTT (5.0 eq.) was added, and the mixture was incubated overnight at 37 °C to form a clear, brownish solution. The pH was set to 3.0 with 10 % HCl following a dialysis (MWCO 1 kDa, RC) against K_2HPO_4/HCl buffer (0.3 mM, pH 5.0) with ascorbic acid/TCEP (each 1 g/15 L) for 3 days and then against K_2HPO_4/HCl buffer (0.3 mM, pH 5.0) for 2 days. The resulting clear solution was freeze dried to give a foamy white solid.

Synthesis of polyethylene glycol-diamine: The amination of PEG was carried out according to Iijima et al. [3] with modifications. Linear PEG (100 g, 1.0 eq.) was dried in vacuo at 110 °C for 2 h, cooled to rt under argon atmosphere and dissolved in dry dichloromethane (900 mL). Triethylamine (3.0 eq.) was added, the mixture cooled to 0 °C and methanesulfonyl chloride (3.0 eq.) added stepwise. The reaction solution was stirred for 1 h at 0 °C and then 4 h at rt after which the solvents were removed with a rotary evaporator at 50 °C and the orange solid dissolved in MilliQ (100 mL). 30 % ammonium hydroxide solution (500 mL) were added, the reaction solution was stirred for 3 days at rt and the remaining ammonia was allowed to evaporate overnight in the fume hood. After a pH adjustment to 9 – 10 with 2 N NaOH and washing the aqueous layer with diethyl ether (3 x 150 mL), the product was extracted with dichloromethane (6 x 150 mL), concentrated in vacuo, and precipitated in cold diethyl ether. The sediment was dried in high vacuum to give a bulky yellowish solid.

Synthesis of polyethylene glycol-diallyl carbamate (2-arm PEG-allyl): Linear PEG-diamine (10 g, 1.0 eq.) was dissolved in MilliQ (100 mL), 2 N NaOH (5.0 eq.) was added and the solution cooled to 0 °C. After the addition of allyl chloroformate (1.6 eq.), the mixture was stirred for 4 h at 0 °C and the aqueous layer was washed with diethyl ether (2 x 100 mL). Extraction was conducted with dichloromethane (5 x 75 mL), the combined organic layers were dried over $MgSO_4$, and filtered. The obtained product solution was concentrated, precipitated in cold diethyl ether, and the sediment dried in vacuo at rt to form white solid.

References

1. Stichler, S.; Böck, T.; Paxton, N.; Bertlein, S.; Levato, R.; Schill, V.; Smolan, W.; Malda, J.; Teßmar, J.; Blunk, T.; et al. Double printing of hyaluronic acid/poly(glycidol) hybrid hydrogels with poly(ε-caprolactone) for MSC chondrogenesis. *Biofabrication* **2017**, *9*, 044108.
2. Vercruysse, K. P.; Marecak, D. M.; Marecek, J. F.; Prestwich, G. D. Synthesis and in Vitro Degradation of New Polyvalent Hydrazide Cross-Linked Hydrogels of Hyaluronic Acid. *Bioconjugate Chem.* **1997**, 8, 686-694.
3. Iijima, M.; Ulkoski, D.; Sakuma, S.; Matsukuma, D.; Nishiyama, N.; Otsuka, H.; Scholz, C. Synthesis of PEGylated poly(amino acid) pentablock copolymers and their self-assembly. *Polym. Int.* **2016**, *65*, 1132-1141.
